# Design, development, and implementation of IsoBank: A centralized repository for isotopic data

**DOI:** 10.1371/journal.pone.0295662

**Published:** 2024-09-06

**Authors:** Oliver N. Shipley, Anna J. Dabrowski, Gabriel J. Bowen, Brian Hayden, Jonathan N. Pauli, Christopher Jordan, Lesleigh Anderson, Adriana Bailey, Clement P. Bataille, Carla Cicero, Hilary G. Close, Craig Cook, Joseph A. Cook, Ankur R. Desai, Jaivime Evaristo, Tim R. Filley, Christine A. M. France, Andrew L. Jackson, Sora Lee Kim, Sebastian Kopf, Julie Loisel, Philip J. Manlick, Jamie M. McFarlin, Bailey C. McMeans, Tamsin C. O’Connell, Suzanne E. Pilaar Birch, Annie L. Putman, Brice X. Semmens, Chris Stantis, Craig A. Stricker, Paul Szejner, Tara L. E. Trammell, Mark D. Uhen, Samantha Weintraub-Leff, Matthew J. Wooller, John W. Williams, Christopher T. Yarnes, Hannah B. Vander Zanden, Seth D. Newsome

**Affiliations:** 1 Department of Biology, University of New Mexico, Albuquerque, New Mexico, United States of America; 2 School of Marine and Atmospheric Sciences, Stony Brook University, Stony Brook, New York, United States of America; 3 Texas Advanced Computing Center, University of Texas at Austin, Austin, Texas, United States of America; 4 Department of Geology and Geophysics, University of Utah, Salt Lake City, Utah, United States of America; 5 Department of Biology, Canadian Rivers Institute, University of New Brunswick, New Brunswick, Canada; 6 Department of Forest and Wildlife Ecology, University of Wisconsin–Madison, Madison, Wisconsin, United States of America; 7 United States Geological Survey Geoscience and Environmental Change Science Center, Denver, Colorado, United States of America; 8 Climate and Space Sciences and Engineering, University of Michigan, Ann Arbor, Michigan, United States of America; 9 Department of Earth and Environmental Sciences, University of Ottawa, Ontario, Canada; 10 Museum of Vertebrate Zoology, University of California, Berkeley, Canada, United States of America; 11 Rosenstiel School of Marine, Atmospheric, and Earth Science, University of Miami, Miami, Florida, United States of America; 12 University of Wyoming Stable Isotope Facility, University of Wyoming, Laramie, Wyoming, United States of America; 13 Department of Atmospheric and Oceanic Sciences, University of Wisconsin-Madison, Madison, Wisconsin, United States of America; 14 Copernicus Institute of Sustainable Development, Utrecht University, Utrecht, The Netherlands; 15 Department of Geography and Environmental Sustainability, University of Oklahoma, Oklahoma, United States of America; 16 Museum Conservation Institute, Smithsonian Institution, Suitland, Maryland, United States of America; 17 School of Natural Sciences, Trinity College Dublin, Dublin, Ireland; 18 Department of Life and Environmental Sciences, University of California, Merced, Merced, California, United States of America; 19 Department of Geological Sciences, University of Colorado, Boulder, Colorado, United States of America; 20 Department of Geography, Texas A&M University, College Station, Texas, United States of America; 21 United States Department of Agriculture, Pacific Northwest Research Station, Juneau, Alaska, United States of America; 22 Department of Geology and Geophysics, University of Wyoming, Laramie, Wyoming, United States of America; 23 Department of Biology, University of Toronto Mississauga, Mississauga, Ontario, Canada; 24 Department of Archaeology, University of Cambridge, Downing Street, United Kingdom; 25 Department of Anthropology and Department of Geography, University of Georgia, Athens, Georgia, United States of America; 26 United States Geological Survey, Utah Water Science Center, West Valley City, Utah, United States of America; 27 Scripps Institution of Oceanography, University of California San Diego, San Diego, California, United States of America; 28 United States Geological Survey, Fort Collins Science Center, Denver, Colorado, United States of America; 29 Bioeconomy and Environment Unit, Natural Resources Institute Finland, Helsinki, Finland; 30 Department of Plant and Soil Sciences, University of Delaware, Newark, Delaware, United States of America; 31 Department of Atmospheric, Oceanic, and Earth Sciences, George Mason University, Fairfax, Vermont, United States of America; 32 National Ecological Observatory Network, Battelle, Boulder, Colorado, United States of America; 33 Alaska Stable Isotope Facility, Water and Environmental Research Center, Institute of Northern Engineering, University of Alaska Fairbanks, Fairbanks, Alaska, United States of America; 34 College of Fisheries and Ocean Sciences, University of Alaska Fairbanks, Fairbanks, Alaska, United States of America; 35 Department of Geography, University of Wisconsin-Madison, Madison, Wisconsin, United States of America; 36 California Davis Stable Isotope Facility, University of California, Davis, California, United States of America; 37 Department of Biology, University of Florida, Gainesville, Florida, United States of America; University of Oklahama Norman Campus: The University of Oklahoma, UNITED STATES

## Abstract

Stable isotope data have made pivotal contributions to nearly every discipline of the physical and natural sciences. As the generation and application of stable isotope data continues to grow exponentially, so does the need for a unifying data repository to improve accessibility and promote collaborative engagement. This paper provides an overview of the design, development, and implementation of IsoBank (www.isobank.org), a community-driven initiative to create an open-access repository for stable isotope data implemented online in 2021. A central goal of IsoBank is to provide a web-accessible database supporting interdisciplinary stable isotope research and educational opportunities. To achieve this goal, we convened a multi-disciplinary group of over 40 analytical experts, stable isotope researchers, database managers, and web developers to collaboratively design the database. This paper outlines the main features of IsoBank and provides a focused description of the core metadata structure. We present plans for future database and tool development and engagement across the scientific community. These efforts will help facilitate interdisciplinary collaboration among the many users of stable isotopic data while also offering useful data resources and standardization of metadata reporting across eco-geoinformatics landscapes.

## 1. Introduction

Stable isotopes are widely used across the physical and natural sciences to trace a wealth of geochemical, environmental, and biological processes [1, 2]. Stable isotope analysis measures the abundance of rarer, heavier forms of an element relative to more abundant, lighter forms (herein ‘isotopes’) such as hydrogen (^2^H/^1^H), carbon (^13^C/^12^C), nitrogen (^15^N/^14^N), oxygen (^18^O/^16^O), sulfur (^34^S/^32^S), and strontium (^87^Sr/^86^Sr). Elemental isotopes vary in mass due to their number of neutrons, which affects reaction rates and bond strengths but not other chemical properties.

Recent improvements in analytical capabilities have made many isotopic measurements routine and cost-effective, such that the number of publications reporting isotopic data have now exceeded the number of publications using gene sequences when GenBank was established [3]. In genetics, the long-term benefits of combining data across disciplines are evinced by the wealth of discoveries enabled by databases such as GenBank [4]. Despite the exponential growth of isotope-based research, the centralized hosting and sharing of isotopic data have not followed the same pattern. Several repositories have emerged to promote isotopic data sharing and centralization, such as IsoMemo (archaeology, ecology, and environmental & life sciences, isomemo.com), IsoArcH (archaeology; [5, 6]), Iso2k (hydrology and climatology; [7, 8]), and the Waterisotopes Database (wiDB; hydrology; [9, 10]). Isotopic data from some fields have been integrated into databases elsewhere with broad proxy data types, such as the Faunal Isotopes within the Neotoma Paleoecology Database [11, 12] or narrowly focused on a particular isotope for example SrIsoMed [13, 14] and iRHUM [15, 16]. These platforms show the potential for data repositories to enable scientific research and new discoveries and have made headway in improving access to isotopic data [3, 17]. However, the stable isotope research community has lacked a globally available, cross-disciplinary data repository with unifying metadata fields. This has limited development of big data isotope-based research and restricted the potential of isotopic information to facilitate systems-based thinking. For example, ecological studies have shown that combining isotopic information from avian feathers and freshwater bodies can permit the tracking of bird migration patterns [18]. But isotopic records from animals and from the hydrosphere are typically preserved in distinct archives whose metadata customs differ substantially. By creating a repository in which isotopic information from diverse disciplines is normalized through a unifying data and metadata template, IsoBank facilitates vast opportunities for novel scientific discovery.

A series of initial concept papers first provided a justification and vision to support the development of an ‘IsoBank’—a centralized, online repository that provides a platform for uploading, hosting, and sharing isotopic data within and across scientific communities [3, 17]. A key challenge identified was unifying the hosting and storage of data across a diverse group of users and analysis types, including the management, long-term storage, quality-assurance-quality-control (QAQC), and standardization of data. Of highest priority and key to the success of a centralized repository was a well-designed metadata structure that adequately described the structure, type, and relationship of data, was guided by the needs and expectations of the scientific community, and promoted effective data re-use and synthesis [3]. Beginning in 2017 with an award from the National Science Foundation (Advances in Biological Informatics), a multi-disciplinary group of analytical experts, stable isotope researchers, database managers, and web developers were convened to lead the design and implementation of IsoBank [19]. IsoBank is now a fully operational open-access repository with an online user interface.

This paper provides an overview of IsoBank’s core features including key aspects of database design and development, metadata structure, data upload and access, and planned future developments. We highlight how IsoBank’s transparent, community-driven development has been key to the database’s design and has allowed for continued refinement of a unified metadata structure that supports a diverse set of disciplines. Finally, we summarize existing and planned community engagements that will help users orient to the database, learn how to upload, and download data, share IsoBank resources, and discuss plans for future data resource/tool development among database users.

## 2. A community-driven approach to database design and development

A series of workshops brought together experts in the measurement, application, and curation of stable isotope data. These workshops identified many challenges associated with developing a database that supports broad and low barrier uptake across isotope-using disciplines and led to the formation of an executive committee for IsoBank. The executive committee comprises experts in five, core sub-disciplines: QAQC, Organismal Biology and Ecology, Archaeology and Paleoecology, Environmental Systems, and Technical Implementation. Members of the executive committee are responsible for the overall vision of IsoBank, and each member leads a discipline-specific sub-committee (DSSC) comprising 5–7 additional experts in their core area.

The DSSCs were responsible for the design of the metadata schema (see Section 4.0), which was refined over a series of bi-annual workshops between March 2017 and November 2021. These workshops were initially hosted in-person, rotating among the institutions of executive committee members, but moved to an online format due to the COVID-19 pandemic. The workshops ensured that all components of the IsoBank data model included support for rigorous QAQC information [20, 21]. This rigor was achieved by working closely with personnel from several well-established stable isotope facilities, such as the University of California Davis and University of Wyoming Stable Isotope Facilities who were central to the QAQC sub-committee.

During the workshops, DSSCs met in break-out sessions to discuss metadata and system needs from their disciplinary or prior shared database development perspectives. These conversations were guided by interactive walkthroughs of the working metadata schema led by the executive committee (*i*.*e*., DSSC leads). Committee-specific online workspaces were created in Google drive that contained editable, shared documents for DSSC members to express needs, write notes, and capture comments. Following breakout sessions, DSSCs rejoined a group session and reported on their discussions and decisions. In this setting, all members had the opportunity to determine where needs overlapped and diverged, and then to reach a consensus in a group conversation.

Following each workshop, the information provided by DSSCs was reviewed, aggregated, and synthesized by the Executive Committee and metadata requirements and system features were prioritized based on the agreements reached in workshops. The Executive Committee used an iterative development process to design basic functionality in the IsoBank system, and then added and adjusted features following input from DSSCs during workshops. The metadata schema including specific metadata field definitions, controlled terms, and logical relations across fields was developed iteratively over several years (see Section 4.0 for more details). Beginning with known standards such as DarwinCore [22, 23], for capturing descriptive metadata and initial input from the Executive Committee, the metadata schema was refined after each workshop and through follow-up communications with each DSSC. Refinements and additions were made to meet the needs across and within specific disciplines. In April of 2021, IsoBank was implemented [19] and members of the Executive Committee designed a series of online and in-person workshops that focused on engagement with the broader community of isotope users. Specifically, these workshops aimed to increase familiarity with the metadata schema and facilitate the upload of new datasets into the repository. These initial engagements were highly successful and began with a 40-person online workshop run through the IsoEcol conference in May 2021 (see [24]).

## 3. Core features of the IsoBank system

The IsoBank system is built on a PostgreSQL database with a web interface developed using the Django framework. Many IsoBank features, such as accessing metadata templates, are publicly accessible and do not require a user account. To be sensitive to researcher challenges with data sharing and open access policies, registered users can choose to upload datasets that are either private (i.e., can only be viewed and queried by the uploader with no embargo period) or can be accessed by any registered user. Storing private data on IsoBank aids users in 1) general data management and/or fulfilling funder requirements for data management and 2) ease of publication and/or making data publicly available at the appropriate time. User registration was initially facilitated by providing an email address and password; however, the system now requires manual approval of new users by administrators to filter non-authentic users (*e*.*g*., bots). Below we provide further information on the features that can be accessed at public, registered user, and administrator levels (Tables 1 & 2).

**Table 1:** Available functionality of IsoBank (www.isobank.org) at public, registered user, and administrator levels.

Webpage Function	Description	Public	Registered User	Administrator
*IsoBank Project Home*	This page provides an overview of recent news and updates related to the IsoBank system, including but not limited to metadata updates, bug fixes, and information on current and upcoming engagement opportunities, such as workshops. We also provide a brief description of IsoBank’s functionality including contact details for technical support.	x	x	x
*IsoBank Guide*	The IsoBank guide provides information related to data upload, including a brief tutorial, accessible links to key metadata documentation, frequently asked questions, and several discipline-specific CSV templates to aid users with data formatting prior to upload. Example templates are currently available for several biological (animal tissue δ^13^C and δ^15^N) and paleoecological (tooth enamel δ^18^O) datasets.	x	x	x
*Project Details*	This page provides access to planning materials and documentation related to community workshops that have taken place since IsoBank’s inception. This includes documentation related to the history, design, and project structure of IsoBank—the latter outlining current members of the executive committee and corresponding DSSCs. A full comprehensive list of current members can be found at www.isobank.org.	x	x	x
*Dataset Statistics*	This tab provides a basic summary detailing the total number of records currently uploaded to IsoBank. Information is ordered based on the metadata schema (see below), including total number of 1) submitted datasets, 2) analysis records, 3) analysis metadata records, 4) measurement records, and 5) measurement metadata records (for full definitions, see Table 2).	x	x	x
*Dataset Lists*	Here registered users can access previously uploaded datasets, which can be downloaded as CSV files. Datasets can be ordered based on the IsoBank ID, submitter, time of submission, file name, title of the dataset, or the dataset description. IsoBank also allows for individual analysis records to be accessed together with metadata supporting each analysis record.	x	x	x
*Data Search*	IsoBank integrates an advanced search interface allowing users to select one or more metadata fields for their search. Once they have chosen the metadata fields, the user enters their preferred search terms. For example, a user can choose to search for data based on the "Collection Date", then specify the dates or date ranges in which they are interested. When adding multiple search terms, the results can be filtered using logical "AND" (data containing all the terms provided by the user) or "OR" (data containing at least one of the terms specified) terms. Results can then be viewed in one of two modes: a tabular view with a list of the data records available, and a map-based view that shows the locations where samples were collected for records containing geographic coordinates. Currently, there is no functionality that supports the download of specific data subsets, or aggregations (*i*.*e*., subsets of data from multiple datasets sharing a unifying metadata field), following data search. Therefore, users must download full datasets as CSV files from the *Dataset Lists* tab.	x	x	x
*Upload Templates*	Registered users can generate data upload templates by selecting elements from the current metadata schema (see Section 4.0). Core metadata fields are automatically selected, and the user can add additional suggested or optional fields applicable to their data type(s). Once all fields have been selected, the user can download their template as a CSV file.		x	x
*Register Lab*	To ensure full transparency and QAQC between analytical laboratories and users of isotopic data, the analytical facility at which measurements were conducted is included as a required metadata field for all IsoBank datasets. While many analytical facilities are already registered, new facilities can be added through the ‘register lab’ tab by providing a unique, character-constrained lab abbreviation (typically no more than four characters) and the full name of the facility. Lab registrations are then reviewed and approved by an IsoBank administrator. Lab abbreviations are entered as controlled terms (*i*.*e*., must include one of several pre-specified terms in a required format) when populating the data upload templates, linking each data record to the lab from which it originated.		x	x
*My Profile*	Once the user’s registration has been created, personal information can be added to their personal profile including first and last names, a contact email address, short bio, and affiliation. This information, while brief, will ensure that data users may contact data submitters with any additional questions or concerns relating to uploaded data.		x	x
*My Data*	Here users are provided with a summary of past uploads ordered by submission date and time. Options are available to delete and reupload datasets if updates are required. From this page, users can access their data in the format of a ‘data list’ (see above), from which individual records and associated metadata can be viewed.		x	x
*Site Administration Tools*	These tools allow administrators to view submitted data and metadata records, manage users and user groups, and update website pages.			x
*Metadata Management Tool*	The Metadata Management tool allows administrators to adjust the metadata schema by defining new metadata fields, adding controlled terms, and indicating where the metadata fields appear on the *Upload Templates* page. New metadata fields are added based on community recommendations and authorized only by the Executive Committee.			x

**Table 2:** Definitions for reported dataset statistics for IsoBank.

Statistic	Definition
Submitted Datasets	The total number of submitted datasets currently stored on IsoBank.
Analysis Records	Total number of records associated with a specific sample of some substance (*e*.*g*., walrus whisker) that can produce multiple measurements (e.g., δ^2^H, δ^15^N, δ^18^O).
Analysis Metadata Records	Unique metadata elements associated with each analysis.
Measurement Records	The total number of records associated with each unique isotope measurements.
Measurement Metadata Records	Unique metadata elements associated with each measurement.

### 3.1 Public access

Public access with no registered user account allows researchers to browse the main features of the repository, access online learning resources, search, and query data, and download hosted datasets (Table 1).

### 3.2 Registered user access

Features associated with uploading data and registering new analytical laboratories require users to create an account that is currently approved by members of the DSSCs and IsoBank development team.

### 3.3 Administrator access

Administrator access is currently limited to the Executive Committee and core technical development team at the Texas Advanced Computing Center (TACC). As IsoBank evolves administration will likely expand to trusted users and consistent contributors to the repository’s future vision (See section 5.0 Current and Planned Developments).

## 4. The metadata schema

### 4.1 Conceptual framework

**Fig 1:**
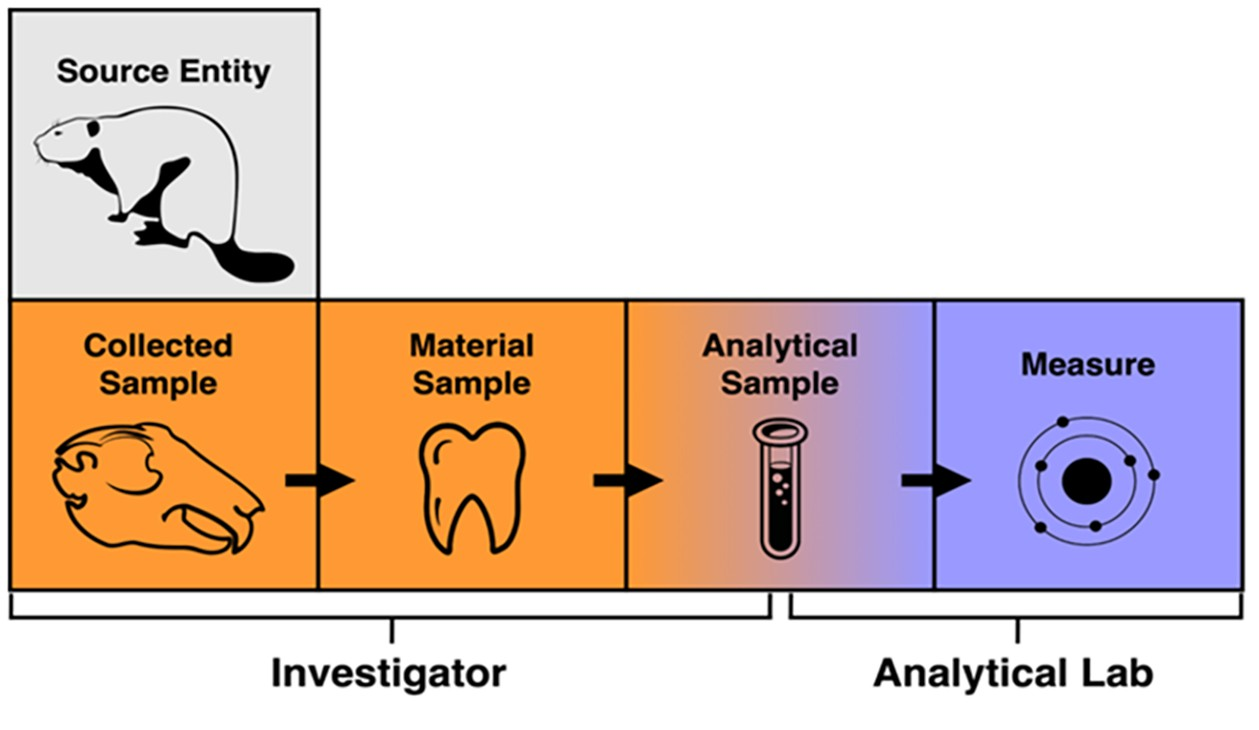
General overview for isotopic analysis of inorganic and organic samples that provided the basis for the metadata schema.

The DSSCs defined the overall metadata requirements for data submitted to IsoBank, which focused on identifying the information that researchers need for stable isotope data discovery and reuse. To guide DSSC group conversations, the Executive Committee created a framework for organizing related information. This framework uses several conceptual entities to describe the context of stable isotope data creation (Fig 1), including a *collected sample*, a *material sample*, and an *analytical sample*, which are defined as:

- a *collected sample* is defined as a physical sample collected in the field or lab from a physical or biological object;
- a *material sample* is the part or whole of the *collected sample* that is prepared for isotopic analysis according to the methodologies of various disciplines;
- an *analytical sample* is the subset of the *material sample*, which is analyzed within an analytical lab.

Sample collection and preparation is conducted or overseen by an individual in the role of an *investigator* (Fig 1). *Material samples* are then processed by an analytical lab, which may or may not be run by the *investigator* (Fig 1). An analytical lab implements additional procedures once it acquires a sample, resulting in an *analytical sample* on which the *measurement* is made. An analytical lab typically then reports measurement data that is quality controlled according to the lab standards (Fig 1).

**Fig 2:**
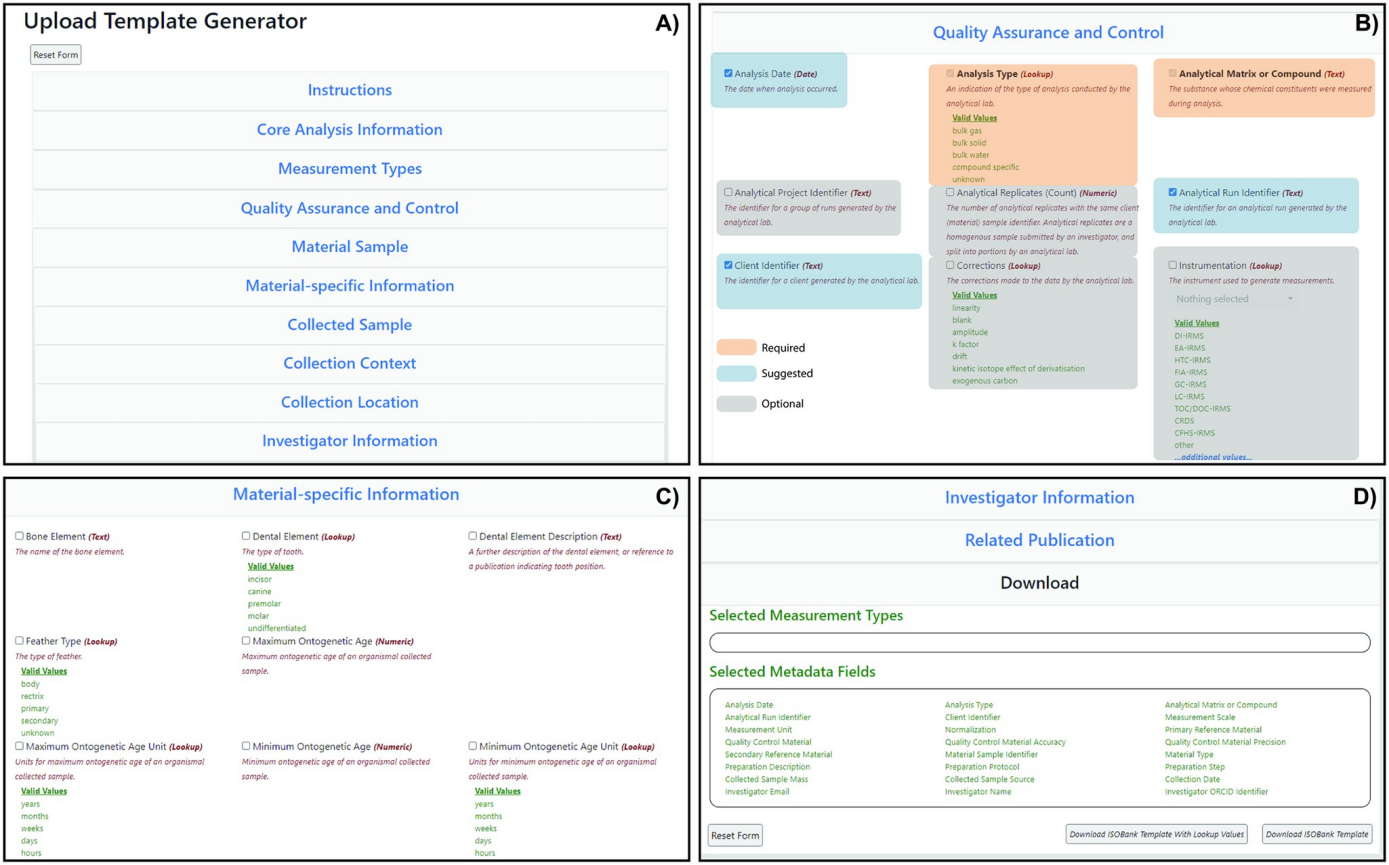
IsoBank template generator used to facilitate data upload. A) Metadata fields are ordered by specific groupings related to the analytical pipeline (Fig 1) and are expanded by clicking on each grey box before manual selection of metadata fields. B) Core/Required (orange), suggested (blue), and optional (gray) metadata fields are selected to maintain uniformity and sufficient levels of QAQC. The user can manually select additional suggested and/or optional fields appropriate to their data types, for which definitions and example terms are highlighted(B). C) For some metadata fields valid values illustrate controlled vocabularies that must be entered for successful upload of data (C). Once all appropriate fields are selected, the user can download the metadata template as a CSV file to begin entering their own data (D).

Based on this context, the IsoBank metadata schema organizes descriptive metadata fields into nine groups associated with the collected, material, and analytical sample entities and their processing (Table 3). As of August 2021, ten groups were used to organize 113 metadata fields within an upload template generator (Fig 2).

**Table 3:** Definitions of nine major metadata groups associated with the IsoBank upload template generator.

Metadata Group	Definition
Core Analysis Information	Information on the analytical facility that performed the isotopic measurements, including ‘Analytical Facility’.
Measurement Types	Information on the specific isotope systems analyzed, for example ‘δ^13^C’, ‘δ^15^N’, ‘%C’, ‘%N’ etc.
Quality Assurance and Control	Information about the analyte or compound destructively analyzed by an analytical lab, as well as quality assurance/control information associated with the reported measurements, including ‘Analysis Type’, ‘Measurement Scale’, and ‘Quality Control Material’.
Material Sample	Information about the material prepared for submission to an analytical lab and the preparation process, including ‘Material Type’ and ‘Preparation Step’.
Material-Specific Information	Information associated with specific material types, for example a sample of the type "tooth" is associated with a ‘Dental Element’ metadata field. Other examples include ‘Sediment Particle Class’ and ‘Water Phase’.
Collected Sample	Information about the physical object or substance acquired by an investigator and its storage prior to preparation, including ‘Collected Sample Mass’ and ‘Collected Sample Preservative’.
Collection Context	Information about the context in which the collected sample was acquired, including ‘Collected Sample Source’ and ‘Collection Site Description’.
Collection Location	Three-dimensional spatial information, primarily associated with the collection of samples in the field, including ‘Collection Latitude’, ‘Collection Longitude’, and ‘Minimum Depth’.
Investigator Information	Information about the individual(s) associated with and responsible for the production of the stable isotope data, including ‘Investigator Email’ and ‘ORCID ID’.
Related Publication	Information about publications related to the data which may provide additional context, including ‘Related Publication Citation’ and ‘Related Publication Identifier’.

### 4.2 Schema design

The IsoBank metadata schema provides a flexible structure for describing isotopic measurements and the context of their creation. Its design attempts to overcome several challenges, including 1) capturing sufficient descriptive information without overburdening data submitters, 2) balancing the needs and expectations of various academic disciplines working with isotopic data, and 3) meeting the requirements of multiple user groups who handle both newly generated and older (i.e., legacy) data. Where possible, the IsoBank schema incorporates metadata fields from existing standards. For example, many fields in the ‘Collection Location’ group follow the DarwinCore metadata standard. In other cases, metadata fields that are unique to stable isotope data and were defined by members of the DSSCs, for example ‘Preparation Step’.

To balance the information needs of data users against the metadata reporting workloads placed on data submitters, IsoBank’s metadata schema emphasizes capturing information essential to the interpretation and reuse of isotopic data, which is pertinent for finding, understanding and comparative reuse (republication) of data available in the IsoBank system. The schema design omits detailed information about physical samples and descriptions of other data derived from the samples, and for example, does not provide other chemical measurements made on samples for which isotopic data are reported. Since these data and metadata may be available in external data systems, the schema includes metadata fields for linking to records elsewhere. For example, the “External Record Provider” and “External Record Identifier” fields in the ‘Collected Sample’ metadata group (Table 3) allow users to refer to sample records in systems like NEON [25], Neotoma [11, 12], or the Arctos data platform for museum collections [26, 27]. IsoBank also connects to external sources to look up additional information. For example, if a user provides a “Scientific Name” for an organism when submitting data, IsoBank will query the Global Biodiversity Information Facility (GBIF, [28]), for additional information and to standardize entries.

In addition to organizing fields, the IsoBank schema also defines how the metadata fields function. Each metadata field has several attributes, including a display name such as “Material Type” and a description that appears on the user interface. Metadata fields will accept a specific type of value, such as free text, numeric, or a term from a controlled list or hierarchy. The value type is then enforced by a validation process when users provide metadata. Importantly, controlled terms (e.g., ‘Analysis Type’, ‘Instrumentation’, and “Material Type’) serve to standardize values within certain metadata fields and improve the effectiveness of each search.

The schema also encodes whether a field is required and the number of values a field can contain. Some fields can contain only one value, while other fields may contain multiple values. IsoBank has only ten required, core metadata fields (Table 4), since researchers from a variety of different disciplines expressed opinions about which metadata fields should be required. These include: ‘Analytical Lab’ (controlled term), ‘Investigator Name’ (free text), ‘Analysis Type’ (controlled term), ‘Analytical Matrix or Compound’ (free text), ‘Measurement Scale’ (controlled term), ‘Measurement Unit’ (controlled term), ‘Material Type’ (controlled term), ‘Preparation Step’ (controlled term), ‘Collected Sample Source’ (controlled term), and ‘Investigator Email’ (free text).

**Table 4:** Definitions associated with IsoBank’s core metadata fields.

Metadata Group	Metadata Field	Template Column Name	Description	Expected Value	Controlled Terms	Deonticity	Conditional Use	Number of Values Allowed
Core Analysis Information	Analytical Lab	*analytical_lab*	The facility at which the isotope measurement was conducted.	Text	Required	Required	None	1
QAQC	Analytical Matrix or Compound	*analytical_matrix*	The substance whose chemical constituents were measured during analysis.	Text	Recommended	Required	None	1+
QAQC	Measurement Scale	*measurement_scale*	Zero-point material that defines the isotope delta-scale to which the measurement is anchored. The international reference scale to which the measurement is being corrected.	Text	Required	Required	None	1
QAQC	Measurement Unit	*measurement_unit*	The unit of measure for a value.	Text	Required	Required	None	1
Material Sample	Material Type	*material_type*	The type of material in a sample submitted for analysis.	Text	Required	Required	None	1
Material Sample	Preparation Step	*preparation_step*	Preparation step(s) performed on the collected sample that resulted in a material sample submitted for isotopic analysis.	Text	Required	Required	None	1+
Collection Context	Collected Sample Source	*collection_source*	An indication of where the collected sample was acquired.	Text	Required	Required	None	1
Investigator Information	Investigator Name	*investigator_name*	The full name of the primary investigator serving as the contact point for the related analysis record.	Text	None	Required	None	1
Investigator Information	Investigator Email	*investigator_email*	The email of the primary investigator serving as the contact point for the related analysis record.	Text	None	Required	None	1

To balance the needs and expectations of various disciplines for specific contextual information, the IsoBank metadata schema allows for logical dependencies between metadata fields. Thus, the value within one field can affect the requirement to use another. Some fields become recommended, and others become required based on the value in another metadata field. For example, choosing “Feather” as the ‘Material Type’ changes the ‘Feather Type’ field from optional to required. The Upload Template Generator uses the logical dependencies defined within the metadata schema to automatically add dependent fields to templates created by users. Because these dependencies are complex, we provide a glossary of all metadata terms, that includes definitions, deonticity, whether fields are considered controlled, their conditional dependencies, and expected values (see S1 File).

### 4.3 Metadata schema management

An administrative interface on the IsoBank website allows administrators (See *Administrator Access*) to manage the metadata schema and field definitions. IsoBank administrators can add metadata fields and adjust their definitions. In addition, administrators can also manage the lists and hierarchies of controlled terms, as well as define dependencies between fields that are conditionally recommended or required. Each metadata field definition includes attributes determining how the field is implemented within the schema (Table 5).

**Table 5:** Definitions of IsoBank metadata attributes that determine how each metadata field is implemented within the current schema.

Metadata Attribute	Definition
*Display name*	The name for the metadata field as displayed on the IsoBank interface.
*Database name*	The name for the metadata field as used within the database.
*Value type*	The type of values accepted by the metadata field, including text, numeric, lookup (list of controlled terms), tree (hierarchy of controlled terms), and date.
*Metadata group*	The group to which the metadata field belongs for organization within the interface: Core Analysis Information, Measurement Types, Quality Assurance and Control, Material Sample, Material-specific Information, Collected Sample, Collection Context, Collection Location, Investigator Information, Related Information.
*Cardinality*	How many values the metadata field will accept: One to many, Zero to many, Zero to one, One and only one.
*Deonticity*	The requirement for using the metadata field: Optional, Recommended, Required, Conditionally Recommended, Conditionally Required.
*Description*.	The text description indicating how the metadata field should be used.
*Examples*.	Example values.
*Detailed description*	A longer description documenting the metadata field.

### 4.4 Data submission and discovery in IsoBank

#### 4.4.1 The upload template generator

As users work through the metadata template generator to create custom data upload templates, they can choose to show or hide groups of metadata fields (Fig 2A). Required metadata fields have grayed out selection boxes and cannot be removed, recommended fields are pre-selected with a blue tick, and optional fields are not pre-selected. (Fig 2B). Each field is followed by a description and valid values (Fig 2C), and changes to the selected metadata fields are reflected at the bottom of the page (Fig 2D). Over time, we envisage the need for the metadata template generator to diminish as scientists begin to share discipline-specific templates with their respective lab groups, colleagues, and collaborators. For example, individual working groups that routinely analyze the same substrate could optimize a template for their specific projects.

#### 4.4.2 Search features, advanced record view, and dataset download

**Fig 3:**
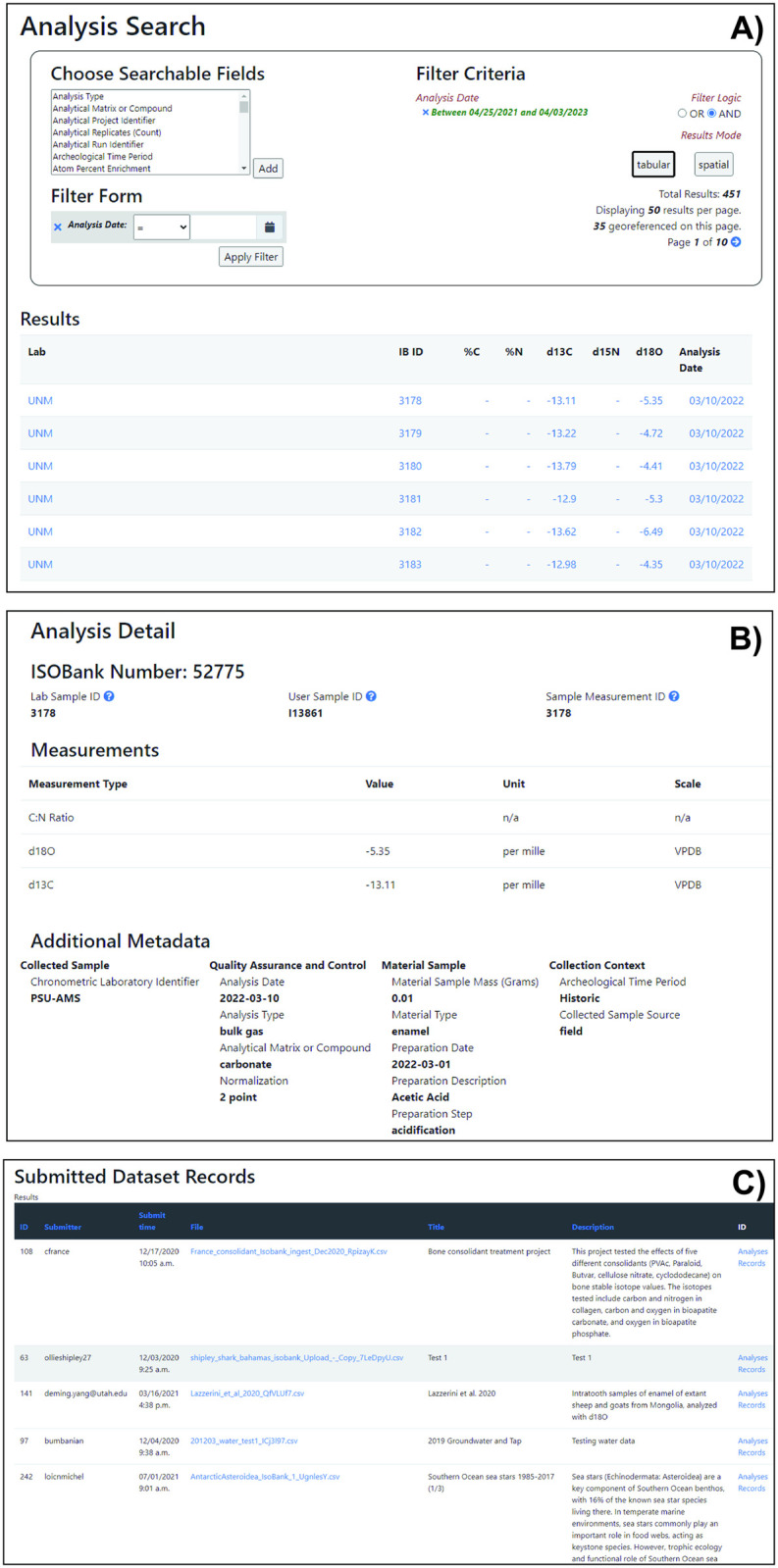
Search and filter functions to explore open access data through IsoBank. Individuals can search based on specific metadata criteria, for which data can be explored using a tabular list view (A) view. Users can access individual record summaries by clicking on the blue text within each list (B), which includes a clickable IsoBank number that defines metadata records associated with a unique sample measurement. These are accessed through the ‘Dataset Lists’ page and can be fully downloaded as CSV files (C).

The IsoBank Data Search interface allows users to find data that has been made publicly available in IsoBank. Users choose which metadata fields to search for and apply filter criteria to find relevant data. For example, choosing “Analysis Date” as a search field enables a user to find data with an exact analysis date, or set additional criteria to find data before, after, or between dates. The search results can be displayed either in list (‘tabular’, Fig 3A) or map view, showing individual data records or records based on sample collection locations, respectively. The user can further explore individual records by clicking on any of the blue highlighted metadata entries, which opens an ‘Analysis Summary’ page corresponding to all metadata fields associated with a specific sample (Fig 3C). This summary page provides an IsoBank ID Number, which corresponds to specific datasets accessed and downloaded as a.csv file through the ‘Dataset List’ tab (Fig 3C).

## 5. Current and planned developments

Here we describe in-progress, planned, and long-term developments of the IsoBank repository. These developments will largely focus on improvements to usability, which can be achieved through increased engagement with the scientific community and achieving a resilient financial model to support long-term sustainability. While submission of competitive grant proposals to funding bodies may provide short-term sustainability, more diverse and innovative models are required to ensure sustainability and resilience to financial instability over the long term. Strategies may include 1) subscription-based pricing, where users or institutions pay fees associated with the hosting of data, 2) integration of value-added services, which may include paying for advanced access to data tools or data tracking (i.e., statistics on how may users are utilizing uploaded datasets), 3) formal assignment of an institutional host that can provide reliable funding in return for credibility, and 4) curated marketing opportunities that require a premium.

### 5.1 Developing community policies

As a community-driven project [29], open-source capabilities are a priority for IsoBank over the long-term. Accordingly, we will develop policies allowing the community to suggest, add, and refine the metadata schema over time. We anticipate this will encourage growth toward shared vocabularies and uniformity in community standards. Further amendments will include developing:

1. Controlled terms that encode shared vocabularies and community standards.
2. IsoBank-wide usage agreement, data licensing, and reuse policies.
3. Data retraction and redaction policies that will support editing of previously uploaded datasets to comply with state-of-the art.
4. Methods for handling duplicate data hosted on IsoBank; this may become an issue for older, legacy data that are not added by the original principal investigator, for example, if they are deceased.
5. Data quality indicators and metadata completeness ratings for published data.
6. Visible versioning for published datasets.
7. Persistent Digital Object Identifiers (DOIs) for each unique dataset, thus providing a platform to increase the citation capacity of data uploaded to the repository. This also provides the opportunity to work with journals, especially those requesting the upload of data to a database prior to publication.

### 5.2 Broader engagement and technological development

**Fig 4:**
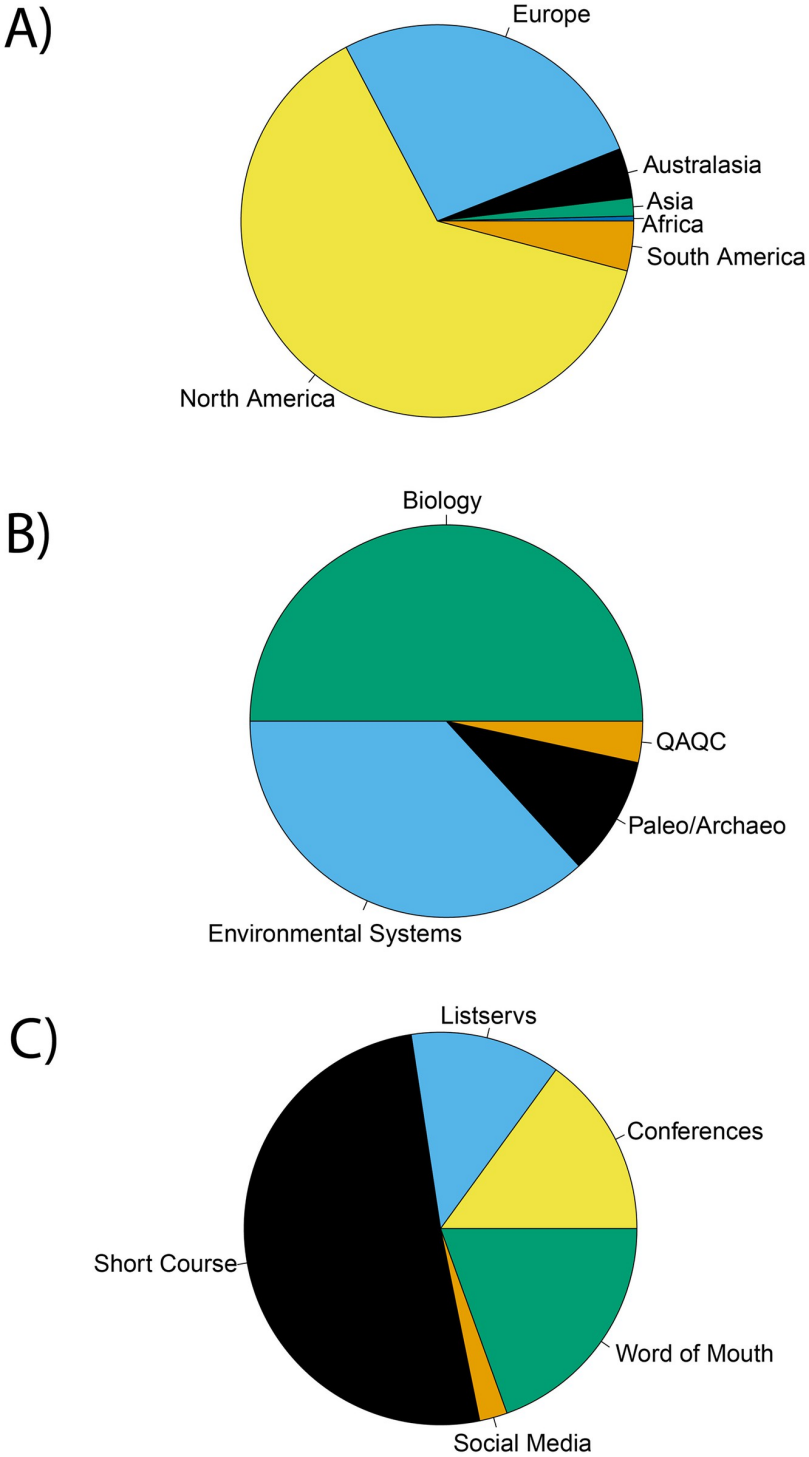
IsoBank community engagement statistics for n = 266 participants by A) geography of affiliation, B) scientific discipline, and C) marketing strategy through which the initial engagement occurred.

IsoBank has focused heavily on community outreach throughout its initial design and implementation phases, largely through a series of online, community-based workshops. Thus far, IsoBank has reached over 260 individuals through workshops and other community engagement initiatives (*e*.*g*., the IsoCamp short course [30]). Of these, a high proportion of workshop participants were based at institutions in North America (63%) and Europe (27%) (Fig 4A), comprising mostly the disciplines of biology (50%) and environmental systems (37% Fig 4B). Workshop participants were engaged through a variety of outreach strategies, including short courses (51%), word-of-mouth (12%), email listservs (12%), social media (2%), and academic conferences (15%) (Fig 4C).

**Fig 5:**
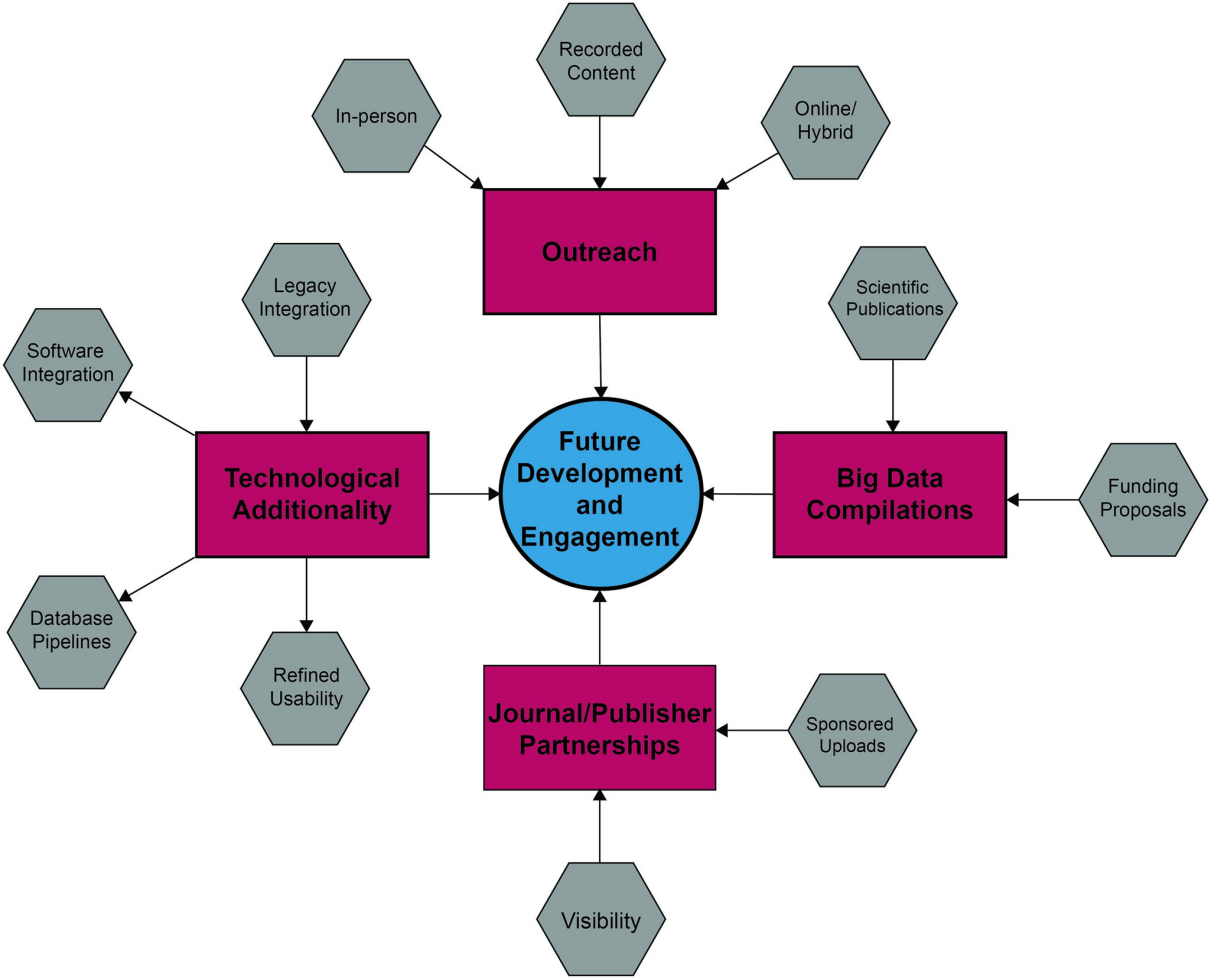
Proposed vision for continued development of community engagement initiatives for IsoBank (maroon boxes), including outreach, journal/publisher partnerships, technological additionality, and facilitation of big data compilations. Gray boxes represent specific activities that improve each engagement initiative.

As IsoBank expands and matures, we will continue to design new engagement opportunities through attendance at professional conferences, symposia, and expansion of available online content such as recorded tutorials and live workshops. We will also enhance an evolving governance model, ensuring constant evaluation and integration of new members into DSSCs, especially regarding community members from currently marginalized and underrepresented groups. This will require looking beyond personal networks. This growth will be facilitated largely by the diversification of outreach strategies, technological advances, journal/publisher partnerships, and big data compilations (Fig 5).

#### 5.2.1 Expanding outreach

We aim to provide a more diverse suite of outreach opportunities that include continued online and in-person workshops focused on orientation associated with data ingest and search. Of particular importance is engagement with scientists at early career stages (e.g., graduate students and postdoctoral researchers), with an interest in big data approaches to science and who will continue to develop new analytical and statistical approaches for isotopic data. IsoBank will continue to be further integrated into existing educational programs and short courses such as IsoCamp [30], SPATIAL [31], and the Survivors Guide to Stable Isotope Ecology [32]. To provide a wide variety of recorded content, tutorials and pre-recorded workshops will also be made available.

#### 5.2.2 Technological advances

We believe that we can increase the efficiency and usability of IsoBank through several key technological advancements that we hope to develop during the next phase of IsoBank. First, developing data transfer between IsoBank and existing database that host isotopic data such as Arctos [26, 27], NEON [25], Neotoma [11, 12], and wiDB [9, 10] and, will facilitate greater uniformity and sharing of larger datasets. Second, growing IsoBank’s capabilities beyond a data repository exclusively would allow for collaborative opportunities with members of the scientific community such as software developers. For example, creating new data transfer protocols for analytical facilities that can directly export newly generated data into an IsoBank-formatted CSV file. Additionally, we envisage the provision of online resources that can host and support the development of novel statistical tools for the correction and analyses of isotopic data. Finally, our goal is to ensure effective integration of older, legacy datasets that cannot currently be uploaded due to missing core metadata information, implement persistent digital object identifiers (DOIs) and increase hosting capabilities.

#### 5.2.3 Journal/publisher partnerships

Scaling of IsoBanks usability would be greatly facilitated through official partnerships with academic publishers and scientific journals, especially as many publishing outlets move toward models that require full transparency and open access to data [33]. In fact, upon acceptance of scientific manuscripts, many journals provide guidance and financial incentives for hosting data through repositories such as DRYAD [34]. Therefore, the development of strong relationships with publishing partners can benefit both IsoBank and the scientific community through advertisement and suggestions for archiving and use of isotopic datasets within a community-defined metadata model. This will also necessitate IsoBank’s compliance with FAIR (Findability, Accessibility, Interoperability, and Reusability, [35]) principles that support effective reuse of scholarly data.

#### 5.2.4 Big data compilations

As IsoBank grows in terms of the type and number of datasets, so will the capacity for cross-disciplinary big data initiatives and integration, which are critical for supporting highly impactful scientific studies of global relevance [36, 37] and development of novel research proposals. Given the high volume of published studies reporting isotope measurements, integration of historical legacy data must be a core focus of IsoBank’s future development. We anticipate that efforts to compile and upload legacy data could be proposed and implemented as funded projects by disciplinary investigators. This would support impactful macroscale research, such as development of novel isoscapes and refinement of global biogeochemical models (e.g., hydrology, carbon cycling, nitrogen cycling etc.).

## 6. Conclusions

The development and implementation of IsoBank now facilitates the centralization of stable isotope data across many disciplines. Through a community-driven approach, we developed a web-based interface that supports the upload and re-use of isotopic data with rigorous reporting of metadata and quality assurance and control information. These data can be used at no cost to the user. The future success of IsoBank depends on scaled engagement with the scientific community and increased technological advances. Over time, we hope that IsoBank becomes a unifying node for the hosting and sharing of isotopic data and associated educational resources, thus allowing for innovative scientific questions to be addressed at large spatial, temporal, and disciplinary scales.

## Supporting information

S1 File IsoBank metadata guide. IsoBAnk metadata guide with full definitions and descriptions of all metadata fields.
(PDF)
