# Supplementary material for: Design, development, and implementation of IsoBank: A centralized repository for isotopic data
Source: PLoS One. 2024 Sep 6;19(9):e0295662. doi: 10.1371/journal.pone.0295662 (PMC11379280; doi:10.1371/journal.pone.0295662)
Supplement: S1 File — IsoBAnk metadata guide with full definitions and descriptions of all metadata fields. (PDF) [file pone.0295662.s001.pdf]

IsoBank Metadata Fields by Group

Last revised: 2021-03-02

Quality Assurance and Control

Analysis Date

CSV Template Column Name: analysis_date
Description: The date and time, date, or year when analysis occurred.
Expected Value: Date: ISO 8601
Controlled Terms: None
Deonticity: Recommended
Conditional Use: None
Number of Values Allowed: 0 – 1

Analysis Type

CSV Template Column Name: analysis_type
Description: An indication of the type of analysis conducted by the analytical lab.
Expected Value: Text: Controlled Term
Controlled Terms: Required:
https://docs.google.com/spreadsheets/d/1HTlFXwLbWjJItnIzds80J4FnErylTr_3mQ04K-hfKKM/edit#gid=1811390642
Deonticity: Required
Conditional Use: None
Number of Values Allowed: 1

Analytical Matrix or Compound

CSV Template Column Name: analytical_matrix
Description: The substance whose chemical constituents were measured during analysis.
Expected Value: Text
Controlled Terms: Recommended:
https://docs.google.com/spreadsheets/d/1HTlFXwLbWjJItnIzds80J4FnErylTr_3mQ04K-hfKKM/edit#gid=696507118
Deonticity: Required
Conditional Use: None
Number of Values Allowed: 1+

Analytical Project Identifier

CSV Template Column Name: analytical_project_id
Description: The identifier for a group of runs generated by the analytical lab.
Expected Value: Text
Controlled Terms: None
Deonticity: Optional
Conditional Use: None
Number of Values Allowed: 0 – 1

Analytical Replicates (Count)

CSV Template Column Name: analytical_replicate_count
Description: The number of analytical replicates with the same client sample identifier/material sample identifier. Analytical replicates are a homogenous sample submitted by an investigator, and split into portions by an analytical lab.

Expected Value: Number
Controlled Terms: None
Deonticity: Optional
Conditional Use: None
Number of Values Allowed: 0 – 1

Analytical Run Identifier

CSV Template Column Name: analytical_run_id
Description: The identifier for an analytical run generated by the analytical lab.
Expected Value: Text
Controlled Terms: None
Deonticity: Recommended
Conditional Use: None
Number of Values Allowed: 0 – 1

Client Identifier

CSV Template Column Name: client_id
Description: The identifier for a client generated by the analytical lab.
Expected Value: Text
Controlled Terms: None
Deonticity: Recommended
Conditional Use: None
Number of Values Allowed: 0 – 1

Corrections

CSV Template Column Name: corrections
Description: The corrections made to the data by the analytical lab.
Expected Value: Text: Controlled Term
Controlled Terms: Required:
https://docs.google.com/spreadsheets/d/1HTlFXwLbWjJItnIzds80J4FnErylTr_3mQ04K-hfKKM/edit#gid=1070140349
Deonticity: Optional
Conditional Use: None
Number of Values Allowed: 0+

Instrumentation

CSV Template Column Name: instrumentation
Description: The instrument used to generate measurements.
Expected Value: Text: Controlled Term
Controlled Terms: Required:
https://docs.google.com/spreadsheets/d/1HTlFXwLbWjJItnIzds80J4FnErylTr_3mQ04K-hfKKM/edit#gid=134926373
Deonticity: Optional
Conditional Use: None
Number of Values Allowed: 0 – 1

Measurement Scale

CSV Template Column Name: measurement_scale
Description: Zero-point material that defines the isotope delta-scale to which the measurement is anchored. The international reference scale to which the measurement is being corrected.
Expected Value: Text: Controlled Term
Controlled Terms: Required:
https://docs.google.com/spreadsheets/d/1HTlFXwLbWjJItnIzds80J4FnErylTr_3mQ04K-hfKKM/edit#gid=994666612
Deonticity: Required

Conditional Use: None
Number of Values Allowed: 1

Measurement Unit

CSV Template Column Name: measurement_unit
Description: The unit of measure for a value.
Expected Value: Text: Controlled Term
Controlled Terms: Required:
https://docs.google.com/spreadsheets/d/1HTlFXwLbWjJItnIzds80J4FnErylTr_3mQ04K-hfKKM/edit# gid=1831406367
Deonticity: Required
Conditional Use: None
Number of Values Allowed: 1

Normalization

CSV Template Column Name: analysis_normalization
Description: The data normalization used by the analytical lab.
Expected Value: Text: Controlled Term
Controlled Terms: Required:
https://docs.google.com/spreadsheets/d/1HTlFXwLbWjJItnIzds80J4FnErylTr_3mQ04K-hfKKM/edit# gid=1875347713
Deonticity: Recommended
Conditional Use: None
Number of Values Allowed: 0+

Other Instrumentation

CSV Template Column Name: other_instrumentation
Description: Additional description of the instrument used to generate measurements.
Expected Value: Text
Controlled Terms: None
Deonticity: Conditionally Recommended
Conditional Use: if Instrumentation: other
Number of Values Allowed: 0 – 1

Primary Reference Material

CSV Template Column Name: primary_reference_material
Description: The internationally certified reference material(s) used to anchor measurements to the international reference scale.
Expected Value: Text
Controlled Terms: Recommended:
https://docs.google.com/spreadsheets/d/1HTlFXwLbWjJItnIzds80J4FnErylTr_3mQ04K-hfKKM/edit# gid=1015443884
Deonticity: Recommended
Conditional Use: None
Number of Values Allowed: 0+

Quality Control Material

CSV Template Column Name: qc_material
Description: The in-house reference material(s) traceable to primary reference materials through internal calibrations. Unused in calibration procedures (e.g."check").
Expected Value: Text
Controlled Terms: None
Deonticity: Recommended
Conditional Use: None
Number of Values Allowed: 0+

Quality Control Material Accuracy

CSV Template Column Name: qc_material_accuracy
Description: The absolute value of the difference between the known and measured values of the quality control material.
Expected Value: Number
Controlled Terms: None
Deonticity: Recommended
Conditional Use: None
Number of Values Allowed: 0+

Quality Control Material Precision

CSV Template Column Name: qc_material_precision
Description: The standard deviation of the reference material and quality control material in an analytical run.
Expected Value: Number
Controlled Terms: None
Deonticity: Recommended
Conditional Use: None
Number of Values Allowed: 0+

Secondary Reference Material

CSV Template Column Name: secondary_reference_material
Description: The tertiary or in-house reference material(s) used to perform calibration procedures.
Expected Value: Text
Controlled Terms: None
Deonticity: Recommended
Conditional Use: None
Number of Values Allowed: 0+

Material Sample

Material Sample Description

CSV Template Column Name: material_sample_description
Description: An additional description of the material sample submitted for analysis.
Expected Value: Text
Controlled Terms: None
Deonticity: Optional
Conditional Use: None
Number of Values Allowed: 0 – 1

Material Sample Identifier

CSV Template Column Name: material_sample_id
Description: The investigator's identifier for a material sample submitted for analysis.
Expected Value: Text
Controlled Terms: None
Deonticity: Recommended
Conditional Use: None
Number of Values Allowed: 0 – 1

Material Sample Mass (Grams)

CSV Template Column Name: material_sample_mass_grams
Description: The mass in grams of the material sample submitted to a lab for analysis.
Expected Value: Number

Controlled Terms: None
Deonticity: Optional
Conditional Use: None
Number of Values Allowed: 0 – 1

Material Sample Size Range Maximum

CSV Template Column Name: material_sample_max_size_range_measurement
Description: Maximum size in the size range of material sample particles prepared for analysis.
Expected Value: Number
Controlled Terms: None
Deonticity: Conditionally Recommended
Conditional Use: if Preparation Step: filtration;sieving
Number of Values Allowed: 0 – 1

Material Sample Size Range Maximum Unit

CSV Template Column Name: material_sample_max_size_range_unit
Description: Units for the maximum size in the size range of material sample particles prepared for analysis.
Expected Value: Text: Controlled Term
Controlled Terms: Required:
https://docs.google.com/spreadsheets/d/1HTlFXwLbWjJItnIzds80J4FnErylTr_3mQ04K-hfKKM/edit#gid=246729225
Deonticity: Conditionally Required
Conditional Use: if Material Sample Size Range Maximum has a value
Number of Values Allowed: 0 – 1

Material Sample Size Range Minimum

CSV Template Column Name: material_sample_min_size_range_measurement
Description: Minimum size in the size range of material sample particles prepared for analysis.
Expected Value: Number
Controlled Terms: None
Deonticity: Conditionally Recommended
Conditional Use: if Preparation Step: filtration;sieving
Number of Values Allowed: 0 – 1

Material Sample Size Range Minimum Unit

CSV Template Column Name: material_sample_min_size_range_unit
Description: Units for the minimum size in the size range of material sample particles prepared for analysis.
Expected Value: Text: Controlled Term
Controlled Terms: Required:
https://docs.google.com/spreadsheets/d/1HTlFXwLbWjJItnIzds80J4FnErylTr_3mQ04K-hfKKM/edit#gid=246729225
Deonticity: Conditionally Required
Conditional Use: if Material Sample Size Range Minimum has a value
Number of Values Allowed: 0 – 1

Material Type

CSV Template Column Name: material_type
Description: The type of material in a sample submitted for analysis.
Expected Value: Text: Controlled Term
Controlled Terms: Required:
https://docs.google.com/spreadsheets/d/1HTlFXwLbWjJItnIzds80J4FnErylTr_3mQ04K-hfKKM/edit#gid=1385252005
Deonticity: Required
Conditional Use: None
Number of Values Allowed: 1

Preparation Date

CSV Template Column Name: preparation_date
Description: The date range, exact date, or date and time when sample preparation occurred. Minimally, a range of years.
Expected Value: Date: ISO 8601
Controlled Terms: None
Deonticity: Conditionally Recommended
Conditional Use: if Preparation Description has a value
Number of Values Allowed: 0 – 1

Preparation Description

CSV Template Column Name: preparation_description
Description: An additional description of the preparation that resulted in a material sample submitted for analysis.
Expected Value: Text
Controlled Terms: None
Deonticity: Recommended
Conditional Use: None
Number of Values Allowed: 0 – 1

Preparation Protocol

CSV Template Column Name: preparation_protocol
Description: A reference to a published sample preparation procedure.
Expected Value: Text
Controlled Terms: None
Deonticity: Recommended
Conditional Use: None
Number of Values Allowed: 0+

Preparation Step

CSV Template Column Name: preparation_step
Description: Preparation step(s) performed on the collected sample that resulted in a material sample submitted for isotopic analysis.
Expected Value: Text: Controlled Term
Controlled Terms: Required:
https://docs.google.com/spreadsheets/d/1HTlFXwLbWjJItnIzds80J4FnErylTr_3mQ04K-hfKKM/edit#gid=0
Deonticity: Required
Conditional Use: None
Number of Values Allowed: 1+

Subsample Datum Point

CSV Template Column Name: subsample_datum
Description: A description of the point on a collected sample from which the subsampling distance was measured.
Expected Value: Text
Controlled Terms: None
Deonticity: Conditionally Recommended
Conditional Use: if Preparation Step: serial subsampling
Number of Values Allowed: 0 – 1

Subsample Position

CSV Template Column Name: subsample_position
Description: A physical or distance measure from the subsample datum point, indicating which portion of a

collected sample was subsampled.
Expected Value: Number
Controlled Terms: None
Deonticity: Conditionally Recommended
Conditional Use: if Preparation Step: serial subsampling
Number of Values Allowed: 0 – 1

Subsample Position Unit

CSV Template Column Name: subsample_position_unit
Description: The unit of measure for the subsample distance from the datum point.
Expected Value: Text
Controlled Terms: None
Deonticity: Conditionally Recommended
Conditional Use: if Preparation Step: serial subsampling
Number of Values Allowed: 0 – 1

Material-specific Information

Bone Element

CSV Template Column Name: bone_element
Description: The name of the bone element.
Expected Value: Text
Controlled Terms: None
Deonticity: Conditionally Recommended
Conditional Use: if Material Type: bone
Number of Values Allowed: 0 – 1

Dental Element

CSV Template Column Name: dental_element
Description: The type of tooth (e.g. incisor, canine).
Expected Value: Text: Controlled Term
Controlled Terms: Required:
https://docs.google.com/spreadsheets/d/1HTlFXwLbWjJItnIzds80J4FnErylTr_3mQ04K-hfKKM/edit#gid=1937550239
Deonticity: Conditionally Recommended
Conditional Use: if Material Type: tooth
Number of Values Allowed: 0 – 1

Dental Element Description

CSV Template Column Name: dental_element_description
Description: A further description or reference to a publication indicating tooth position (e.g., lower left premolar 3 or anterior/lateral)
Expected Value: Text
Controlled Terms: None
Deonticity: Conditionally Recommended
Conditional Use: if Material Type: tooth
Number of Values Allowed: 0 – 1

Feather Type

CSV Template Column Name: feather_type
Description: TBD
Expected Value: Text: Controlled Term
Controlled Terms: Required:
https://docs.google.com/spreadsheets/d/1HTlFXwLbWjJItnIzds80J4FnErylTr_3mQ04K-

hfKKM/edit#gid=1607855185
Deonticity: Conditionally Required
Conditional Use: if Material Type: feather
Number of Values Allowed: 0+

Maximum Ontogenetic Age

CSV Template Column Name: max_ontogenetic_age
Description: Maximum ontogenetic age of an organismal collected sample.
Expected Value: Number
Controlled Terms: None
Deonticity: Optional
Conditional Use: None
Number of Values Allowed: 0 – 1

Maximum Ontogenetic Age Unit

CSV Template Column Name: max_ontogenetic_age_unit
Description: Units for maximum ontogenetic age of an organismal collected sample.
Expected Value: Text: Controlled Term
Controlled Terms: Required:
https://docs.google.com/spreadsheets/d/1HTlFXwLbWjJItnIzds80J4FnErylTr_3mQ04K-hfKKM/edit#gid=549651087
Deonticity: Conditionally Required
Conditional Use: if Maximum Ontogenetic Age has a value
Number of Values Allowed: 0 – 1

Minimum Ontogenetic Age

CSV Template Column Name: min_ontogenetic_age
Description: Minimum ontogenetic age of an organismal collected sample.
Expected Value: Number
Controlled Terms: None
Deonticity: Optional
Conditional Use: None
Number of Values Allowed: 0 – 1

Minimum Ontogenetic Age Unit

CSV Template Column Name: min_ontogenetic_age_unit
Description: Units for minimum ontogenetic age of an organismal collected sample.
Expected Value: Text: Controlled Term
Controlled Terms: Required:
https://docs.google.com/spreadsheets/d/1HTlFXwLbWjJItnIzds80J4FnErylTr_3mQ04K-hfKKM/edit#gid=549651087
Deonticity: Conditionally Required
Conditional Use: if Minimum Ontogenetic Age has a value
Number of Values Allowed: 0 – 1

Ontogenetic Age Estimate

CSV Template Column Name: ontogenetic_age
Description: The ontogenetic age estimate of an organismal collected sample.
Expected Value: Number
Controlled Terms: None
Deonticity: Conditionally Recommended
Conditional Use: if Material Type: organism
Number of Values Allowed: 0 – 1

Ontogenetic Age Unit

CSV Template Column Name: ontogenetic_age_unit
Description: The unit of measure for the ontogenetic age estimate of an organismal collected sample.
Expected Value: Text: Controlled Term
Controlled Terms: Required:
https://docs.google.com/spreadsheets/d/1HTlFXwLbWjJItnIzds80J4FnErylTr_3mQ04K-hfKKM/edit#gid=549651087
Deonticity: Conditionally Required
Conditional Use: if Ontogenetic Age Estimate has a value
Number of Values Allowed: 0 – 1

Ontogenetic/Life Stage

CSV Template Column Name: life_stage
Description: Life or ontogenetic stage of an organismal collected sample.
Expected Value: Text
Controlled Terms: Recommended:
https://docs.google.com/spreadsheets/d/1HTlFXwLbWjJItnIzds80J4FnErylTr_3mQ04K-hfKKM/edit#gid=12146707
Deonticity: Conditionally Recommended
Conditional Use: if Material Type: organism
Number of Values Allowed: 0 – 1

Organism Sex

CSV Template Column Name: organism_sex
Description: Sex of the organism collected, or organism from which a sample was collected.
Expected Value: Text: Controlled Term
Controlled Terms: Required:
https://docs.google.com/spreadsheets/d/1HTlFXwLbWjJItnIzds80J4FnErylTr_3mQ04K-hfKKM/edit#gid=2142783624
Deonticity: Optional
Conditional Use: None
Number of Values Allowed: 0 – 1

Reproductive Condition

CSV Template Column Name: reproductive_condition
Description: Reproductive condition of the organism collected, or organism from which a sample was collected.
Expected Value: Text: Controlled Term
Controlled Terms: Required:
https://docs.google.com/spreadsheets/d/1HTlFXwLbWjJItnIzds80J4FnErylTr_3mQ04K-hfKKM/edit#gid=310231711
Deonticity: Optional
Conditional Use: None
Number of Values Allowed: 0 – 1

Reproductive Condition Description

CSV Template Column Name: reproductive_condition_description
Description: An additional description of the reproductive condition of the organism collected, or organism from which a sample was collected.
Expected Value: Text
Controlled Terms: None
Deonticity: Conditionally Required
Conditional Use: if Reproductive Condition: breeding
Number of Values Allowed: 0 – 1

Scientific Name

CSV Template Column Name: scientific_name
Description: The full scientific name at the lowest Taxonomic Rank that can be determined. This field is also used to automatically find the Global Biodiversity Information Facility (GBIF) Taxon ID for the sample.
Expected Value: Text
Controlled Terms: None
Deonticity: Conditionally Recommended
Conditional Use: if Material Type: organism
Number of Values Allowed: 0+

Sediment Particle Class

CSV Template Column Name: sediment_particle_class
Description: Catagorical class based on size or settling velocity: Sediment particles are classified into six general categories: clay, silt, sand, gravel, cobbles, and boulders.
Expected Value: Text: Controlled Term
Controlled Terms: Required:
https://docs.google.com/spreadsheets/d/1HTlFXwLbWjJItnIzds80J4FnErylTr_3mQ04K-hfKKM/edit# gid=1439076994
Deonticity: Conditionally Recommended
Conditional Use: if Material Type: sediment
Number of Values Allowed: 0 – 1

Sediment Source

CSV Template Column Name: sediment_source
Description: The source of sediment.
Expected Value: Text: Controlled Term
Controlled Terms: Required:
https://docs.google.com/spreadsheets/d/1HTlFXwLbWjJItnIzds80J4FnErylTr_3mQ04K-hfKKM/edit# gid=639510883
Deonticity: Optional
Conditional Use: None
Number of Values Allowed: 0 – 1

Soil Description

CSV Template Column Name: soil_description
Description: A description of the soil.
Expected Value: Text
Controlled Terms: None
Deonticity: Optional
Conditional Use: None
Number of Values Allowed: 0 – 1

Soil Horizon

CSV Template Column Name: soil_horizon
Description: The soil horizon (e.g., O, A, B).
Expected Value: Text: Controlled Term
Controlled Terms: Required:
https://docs.google.com/spreadsheets/d/1HTlFXwLbWjJItnIzds80J4FnErylTr_3mQ04K-hfKKM/edit# gid=1815889321
Deonticity: Optional
Conditional Use: None
Number of Values Allowed: 0 – 1

Taxonomic Rank

CSV Template Column Name: taxon_rank
Description: Lowest taxonomic rank that can be determined. The taxonomic rank of the most specific name in the Scientific Name.
Expected Value: Text: Controlled Term
Controlled Terms: Required:
https://docs.google.com/spreadsheets/d/1HTlFXwLbWjJItnIzds80J4FnErylTr_3mQ04K-hfKKM/edit# gid=1311544189
Deonticity: Conditionally Recommended
Conditional Use: if Material Type: organism
Number of Values Allowed: 0+

Water Phase

CSV Template Column Name: water_phase
Description: The phase of the water.
Expected Value: Text: Controlled Term
Controlled Terms: Required:
https://docs.google.com/spreadsheets/d/1HTlFXwLbWjJItnIzds80J4FnErylTr_3mQ04K-hfKKM/edit# gid=1526783427
Deonticity: Optional
Conditional Use: None
Number of Values Allowed: 0 – 1

Water Source

CSV Template Column Name: water_source
Description: The source of water.
Expected Value: Text: Controlled Term
Controlled Terms: Required:
https://docs.google.com/spreadsheets/d/1HTlFXwLbWjJItnIzds80J4FnErylTr_3mQ04K-hfKKM/edit# gid=855656453
Deonticity: Optional
Conditional Use: None
Number of Values Allowed: 0 – 1

Collected Sample

Chronometric Age Measurement (BP)

CSV Template Column Name: chronometric_measurement
Description: The chronometric age reported as the minimum of an age estimate in years before the present (BP).
Expected Value: Number
Controlled Terms: None
Deonticity: Optional
Conditional Use: None
Number of Values Allowed: 0 – 1

Chronometric Age Standard Deviation

CSV Template Column Name: chronometric_standard_deviation
Description: The standard deviation on the the chronometric age measurement.
Expected Value: Number
Controlled Terms: None
Deonticity: Conditionally Recommended
Conditional Use: if Chronometric Age Measurement (BP) has a value
Number of Values Allowed: 0 – 1

Chronometric Dating Method

CSV Template Column Name: chronometric_method
Description: The method of dating used for the chronometric age measurement.
Expected Value: Text
Controlled Terms: Recommended:
https://docs.google.com/spreadsheets/d/1HTlFXwLbWjJItnIzds80J4FnErylTr_3mQ04K-hfKKM/edit# gid=1873062020
Deonticity: Conditionally Required
Conditional Use: if Chronometric Age Measurement (BP) has a value
Number of Values Allowed: 0 – 1

Chronometric Laboratory Identifier

CSV Template Column Name: chronometric_lab_id
Description: The unique identifier (or code) for the chronometric dating laboratory.
Expected Value: Text
Controlled Terms: None
Deonticity: Optional
Conditional Use: None
Number of Values Allowed: 0 – 1

Collected Sample Identifier

CSV Template Column Name: collected_sample_id
Description: The identifier(s) used by an investigator for the collected sample.
Expected Value: Text
Controlled Terms: None
Deonticity: Optional
Conditional Use: None
Number of Values Allowed: 0+

Collected Sample Mass

CSV Template Column Name: collected_sample_mass
Description: The mass of a collected sample.
Expected Value: Number
Controlled Terms: None
Deonticity: Recommended
Conditional Use: None
Number of Values Allowed: 0 – 1

Collected Sample Mass Unit

CSV Template Column Name: collected_sample_mass_unit
Description: The unit of measure for the mass of a collected sample.
Expected Value: Text: Controlled Term
Controlled Terms: Required
Deonticity: Conditionally Required
Conditional Use: if Collected Sample Mass has a value
Number of Values Allowed: 0 – 1

Collected Sample Preservative

CSV Template Column Name: collected_sample_preservative
Description: The preservative(s) used on the collected sample by the investigator.
Expected Value: Text: Controlled Term
Controlled Terms: Required:
https://docs.google.com/spreadsheets/d/1HTlFXwLbWjJItnIzds80J4FnErylTr_3mQ04K-hfKKM/edit# gid=2128818423
Deonticity: Optional
Conditional Use: None

Number of Values Allowed: 0+

Collected Sample Preservative Description

CSV Template Column Name: collected_sample_preservative_description
Description: An additional description of the preservative(s) used on the collected sample by the investigator.
Expected Value: Text
Controlled Terms: None
Deonticity: Conditionally Required
Conditional Use: if Collected Sample Preservative: other preservative
Number of Values Allowed: 0 – 1

Collected Sample Repository Name

CSV Template Column Name: collected_sample_repository_name
Description: The name of the repository for long-term storage of a sample (or parts of a sample) collected in the field by the investigator.
Expected Value: Text
Controlled Terms: None
Deonticity: Conditionally Recommended
Conditional Use: if Collected Sample Repository Type has a value
Number of Values Allowed: 0 – 1

Collected Sample Repository Type

CSV Template Column Name: collected_sample_repository_type
Description: The type of repository for long-term storage of a sample (or parts of a sample) collected in the field by the investigator.
Expected Value: Text: Controlled Term
Controlled Terms: Required:
https://docs.google.com/spreadsheets/d/1HTlFXwLbWjJItnIzds80J4FnErylTr_3mQ04K-hfKKM/edit#gid=1576248359
Deonticity: Optional
Conditional Use: None
Number of Values Allowed: 0 – 1

Collected Sample Size Measurement

CSV Template Column Name: collected_sample_size_measurement
Description: The numeric measure for a parameter of the collected sample that indicates the size of the collected sample.
Expected Value: Number
Controlled Terms: None
Deonticity: Optional
Conditional Use: None
Number of Values Allowed: 0+

Collected Sample Size Parameter

CSV Template Column Name: collected_sample_size_parameter
Description: A description of the paramater measured to indicate the size of the collected sample. For example, “width” or “wing length”.
Expected Value: Text
Controlled Terms: None
Deonticity: Conditionally Required
Conditional Use: if Collected Sample Size Measurement has a value
Number of Values Allowed: 0+

Collected Sample Size Range Maximum

CSV Template Column Name: collected_sample_max_size_range_measurement
Description: Maximum size in the size range of collected sample particles.
Expected Value: Number
Controlled Terms: None
Deonticity: Conditionally Recommended
Conditional Use: if Material Type: wet deposition particles;water particulate;sediment;soil;airborne aerosol;plankton;particulate organic matter
Number of Values Allowed: 0 – 1

Collected Sample Size Range Maximum Unit

CSV Template Column Name: collected_sample_max_size_range_unit
Description: Units for maximum size of collected particles.
Expected Value: Text: Controlled Term
Controlled Terms: Required:
https://docs.google.com/spreadsheets/d/1HTlFXwLbWjJItnIzds80J4FnErylTr_3mQ04K-hfKKM/edit#gid=246729225
Deonticity: Conditionally Required
Conditional Use: if Collected Sample Size Range Maximum has a value
Number of Values Allowed: 0 – 1

Collected Sample Size Range Minimum

CSV Template Column Name: collected_sample_min_size_range_measurement
Description: Minimum size in the size range of collected sample particles.
Expected Value: Number
Controlled Terms: None
Deonticity: Conditionally Recommended
Conditional Use: if Material Type: water particulate;wet deposition particles;airborne aerosol;sediment;soil;plankton;particulate organic matter
Number of Values Allowed: 0 – 1

Collected Sample Size Range Minimum Unit

CSV Template Column Name: collected_sample_min_size_range_unit
Description: Units for minimum size of collected particles.
Expected Value: Text: Controlled Term
Controlled Terms: Required:
https://docs.google.com/spreadsheets/d/1HTlFXwLbWjJItnIzds80J4FnErylTr_3mQ04K-hfKKM/edit#gid=246729225
Deonticity: Conditionally Required
Conditional Use: if Collected Sample Size Range Minimum has a value
Number of Values Allowed: 0 – 1

Collected Sample Size Unit

CSV Template Column Name: collected_sample_size_unit
Description: The unit of measure for the size of a collected sample.
Expected Value: Text: Controlled Term
Controlled Terms: Required
Deonticity: Conditionally Required
Conditional Use: if Collected Sample Size Measurement has a value
Number of Values Allowed: 0+

Collected Sample Storage Container

CSV Template Column Name: collected_sample_storage_container
Description: The type of storage container(s) used for the collected sample by the investigator.
Expected Value: Text: Controlled Term
Controlled Terms: Required:

https://docs.google.com/spreadsheets/d/1HTlFXwLbWjJItnIzds80J4FnErylTr_3mQ04K-hfKKM/edit# gid=1921310866
Deonticity: Optional
Conditional Use: None
Number of Values Allowed: 0+

Collected Sample Storage Container Description

CSV Template Column Name: collected_sample_storage_container_description
Description: An additional description of the type of storage container(s) used for the collected sample by the investigator.
Expected Value: Text
Controlled Terms: None
Deonticity: Conditionally Recommended
Conditional Use: if Collected Sample Storage Container: other container
Number of Values Allowed: 0 – 1

Collected Sample Storage Temperature (Celsius)

CSV Template Column Name: collected_sample_storage_temperature_celsius
Description: The temperature in Celsius at which the sample material was stored by the investigator.
Expected Value: Number
Controlled Terms: None
Deonticity: Optional
Conditional Use: None
Number of Values Allowed: 0 – 1

External Record Identifier

CSV Template Column Name: external_record_id
Description: An identifier to locate a record about the collected sample in an external service.
Expected Value: Text
Controlled Terms: Recommended: **https://docs.google.com/spreadsheets/d/13G5x-eIsKTxkUTcID0j1qtPtmWlWHSnu2VAmytTfGfA/edit?usp=sharing**
Deonticity: Conditionally Recommended
Conditional Use: if Collected Sample Source: existing collection
Number of Values Allowed: 0 – 1

External Record Provider

CSV Template Column Name: external_record_provider
Description: An external service managing information about a collected sample and its stewardship.
Expected Value: Text: Controlled Term
Controlled Terms: Required: **https://docs.google.com/spreadsheets/d/13G5x-eIsKTxkUTcID0j1qtPtmWlWHSnu2VAmytTfGfA/edit?usp=sharing**
Deonticity: Conditionally Recommended
Conditional Use: if Collected Sample Source has a value
Number of Values Allowed: 0 – 1

External Sample Identifier

CSV Template Column Name: external_sample_id
Description: An identifier for the collected sample created by an external service, for example the International Geo Sample Number (ISGN).
Expected Value: Text
Controlled Terms: Recommended: **https://docs.google.com/spreadsheets/d/13G5x-eIsKTxkUTcID0j1qtPtmWlWHSnu2VAmytTfGfA/edit?usp=sharing**
Deonticity: Conditionally Required
Conditional Use: if External Sample Identifier Provider has a value
Number of Values Allowed: 0 – 1

External Sample Identifier Provider

CSV Template Column Name: external_sample_id_provider
Description: An external service that mints unique identifiers for physical samples collected by researchers, for example the International Geo Sample Number (ISGN).
Expected Value: Text: Controlled Term
Controlled Terms: Required: **https://docs.google.com/spreadsheets/d/13G5x-eIsKTxkUTcID0j1qtPtmWlWHSnu2VAmytTfGfA/edit?usp=sharing**
Deonticity: Conditionally Required
Conditional Use: if External Sample Identifier has a value
Number of Values Allowed: 0 – 1

Collection Context

Archeological Time Period

CSV Template Column Name: archeological_period
Description: The name of the archaeological time period.
Expected Value: Text
Controlled Terms: None
Deonticity: Optional
Conditional Use: None
Number of Values Allowed: 0+

Atom Percent Enrichment

CSV Template Column Name: atom_percent_enrichment
Description: Percent enrichment of natural abundance of reference material. Used if Experimental Manipulation includes Artificial Enrichment.
Expected Value: Number
Controlled Terms: None
Deonticity: Conditionally Required
Conditional Use: if Experimental Manipulation: artificial enrichment
Number of Values Allowed: 0 – 1

Biome

CSV Template Column Name: biome
Description: The naturally occurring community of flora and fauna occupying the habitat.
Expected Value: Text
Controlled Terms: None
Deonticity: Optional
Conditional Use: None
Number of Values Allowed: 0+

Collected Sample Source

CSV Template Column Name: collection_source
Description: An indication of where the collected sample was acquired.
Expected Value: Text: Controlled Term
Controlled Terms: Required:
https://docs.google.com/spreadsheets/d/1HTlFXwLbWjJItnIzds80J4FnErylTr_3mQ04K-hfKKM/edit#gid=903030859
Deonticity: Required
Conditional Use: None
Number of Values Allowed: 1

Collection Date

CSV Template Column Name: collection_date
Description: The date or date and time when sample collection occurred.
Expected Value: Date: ISO 8601
Controlled Terms: None
Deonticity: Recommended
Conditional Use: if Collected Sample Source: field
Number of Values Allowed: 0 – 1

Collection Description

CSV Template Column Name: collection_description
Description: An additional description of the sample collection event.
Expected Value: Text
Controlled Terms: None
Deonticity: Optional
Conditional Use: None
Number of Values Allowed: 0 – 1

Collection Protocol

CSV Template Column Name: collection_protocol
Description: Reference to a published sample collection protocol.
Expected Value: Text
Controlled Terms: None
Deonticity: Optional
Conditional Use: if Collected Sample Source: field
Number of Values Allowed: 0 – 1

Collection Site Description

CSV Template Column Name: collection_site_description
Description: An additional description of the collection site.
Expected Value: Text
Controlled Terms: None
Deonticity: Optional
Conditional Use: None
Number of Values Allowed: 0 – 1

Collection Site Identifier

CSV Template Column Name: collection_site_id
Description: The investigator's identifier for a collection site.
Expected Value: Text
Controlled Terms: None
Deonticity: Optional
Conditional Use: None
Number of Values Allowed: 0+

Culture Group

CSV Template Column Name: culture_group
Description: Archaeological or anthropological category describing the context in which a sample was collected.
Expected Value: Text
Controlled Terms: None
Deonticity: Optional
Conditional Use: None
Number of Values Allowed: 0 – 1

Experimental Design

CSV Template Column Name: experimental_design
Description: Reference to a published experimental design.
Expected Value: Text
Controlled Terms: None
Deonticity: Optional
Conditional Use: None
Number of Values Allowed: 0 – 1

Experimental Manipulation

CSV Template Column Name: experimental_manipulation
Description: The experimental manipulation(s) performed, or none.
Expected Value: Text: Controlled Term
Controlled Terms: Required:
https://docs.google.com/spreadsheets/d/1HTlFXwLbWjJItnIzds80J4FnErylTr_3mQ04K-hfKKM/edit# gid=1925389145
Deonticity: Conditionally Recommended
Conditional Use: if Collected Sample Source: field
Number of Values Allowed: 0+

Experimental Manipulation Description

CSV Template Column Name: experimental_manipulation_description
Description: An additional description of the experimental manipulation.
Expected Value: Text
Controlled Terms: None
Deonticity: Conditionally Required
Conditional Use: if Experimental Manipulation: other
Number of Values Allowed: 0 – 1

Geological Time Period

CSV Template Column Name: geological_period
Description: The name of the geological time period.
Expected Value: Text
Controlled Terms: None
Deonticity: Optional
Conditional Use: None
Number of Values Allowed: 0+

Stratigraphic Layer

CSV Template Column Name: stratigraphic_layer
Description: The most specific stratigraphic layer or unit possible, either formally identified or not.
Expected Value: Text
Controlled Terms: None
Deonticity: Optional
Conditional Use: None
Number of Values Allowed: 0 – 1

Verbatim Collection Date

CSV Template Column Name: verbatim_collection_date
Description: An estimated date of collection, if exact date(s) is unknown.
Expected Value: Text
Controlled Terms: None
Deonticity: Optional

Conditional Use: if Collection Date has a value
Number of Values Allowed: 0 – 1

Collection Location

Collection Latitude (Decimal Degrees)

CSV Template Column Name: collection_decimal_latitude
Description: The geographic latitude (in decimal degrees, using the spatial reference system given in geodeticDatum) of the geographic center of a Location. Positive values are north of the Equator, negative values are south of it. Legal values lie between -90 and 90, inclusive.
Expected Value: Number: -90 – 90
Controlled Terms: None
Deonticity: Conditionally Recommended
Conditional Use: if Collected Sample Source: field
Number of Values Allowed: 0 – 1

Collection Locality

CSV Template Column Name: collection_locality
Description: The specific description of the place. A helpful reference may be: **https://www.geonames.org**
Expected Value: Text
Controlled Terms: None
Deonticity: Optional
Conditional Use: None
Number of Values Allowed: 0 – 1

Collection Longitude (Decimal Degrees)

CSV Template Column Name: collection_decimal_longitude
Description: The geographic longitude (in decimal degrees, using the spatial reference system given in geodeticDatum) of the geographic center of a Location. Positive values are east of the Greenwich Meridian, negative values are west of it. Legal values lie between -180 and 180, inclusive.
Expected Value: Number: -180 – 180
Controlled Terms: None
Deonticity: Conditionally Recommended
Conditional Use: if Collected Sample Source: field
Number of Values Allowed: 0 – 1

Geodetic Datum

CSV Template Column Name: geodetic_datum
Description: The ellipsoid, geodetic datum, or spatial reference system (SRS) upon which the geographic coordinates given in Latitude and Longitude as based.
Expected Value: Text
Controlled Terms: None
Deonticity: Conditionally Recommended
Conditional Use: if Collection Latitude (Decimal Degrees);Collection Longitude (Decimal Degrees) has a value
Number of Values Allowed: 0 – 1

Maximum Depth (Meters)

CSV Template Column Name: max_depth_meters
Description: The greater depth of a range of depth below the local surface, in meters.
Expected Value: Number
Controlled Terms: None
Deonticity: Optional
Conditional Use: None

Number of Values Allowed: 0 – 1

Maximum Distance Above Surface (Meters)

CSV Template Column Name: max_distance_above_surface_meters
Description: The greater distance in a range of distance from a reference surface in the vertical direction, in meters. Use positive values for locations above the surface, negative values for locations below. If depth measures are given, the reference surface is the location given by the depth, otherwise the reference surface is the location given by the elevation.
Expected Value: Number
Controlled Terms: None
Deonticity: Optional
Conditional Use: None
Number of Values Allowed: 0 – 1

Maximum Elevation (Meters)

CSV Template Column Name: max_elevation_meters
Description: The upper limit of the range of elevation (altitude, usually above sea level), in meters.
Expected Value: Number
Controlled Terms: None
Deonticity: Optional
Conditional Use: None
Number of Values Allowed: 0 – 1

Minimum Depth (Meters)

CSV Template Column Name: min_depth_meters
Description: The lesser depth of a range of depth below the local surface, in meters.
Expected Value: Number
Controlled Terms: None
Deonticity: Optional
Conditional Use: None
Number of Values Allowed: 0 – 1

Minimum Distance Above Surface (Meters)

CSV Template Column Name: min_distance_above_surface_meters
Description: The lesser distance in a range of distance from a reference surface in the vertical direction, in meters. Use positive values for locations above the surface, negative values for locations below. If depth measures are given, the reference surface is the location given by the depth, otherwise the reference surface is the location given by the elevation.
Expected Value: Number
Controlled Terms: None
Deonticity: Optional
Conditional Use: None
Number of Values Allowed: 0 – 1

Minimum Elevation (Meters)

CSV Template Column Name: min_elevation_meters
Description: The lower limit of the range of elevation (altitude, usually above sea level), in meters.
Expected Value: Number
Controlled Terms: None
Deonticity: Optional
Conditional Use: None
Number of Values Allowed: 0 – 1

Investigator Information

Investigator Email

CSV Template Column Name: investigator_email
Description: The email of the primary investigator serving as the contact point for the related analysis record.
Expected Value: Text
Controlled Terms: None
Deonticity: Required
Conditional Use: None
Number of Values Allowed: 1

Investigator Name

CSV Template Column Name: investigator_name
Description: The full name of the primary investigator serving as the contact point for the related analysis record.
Expected Value: Text
Controlled Terms: None
Deonticity: Required
Conditional Use: None
Number of Values Allowed: 1

Investigator ORCID Identifier

CSV Template Column Name: investigator_orcid
Description: The ORCID Identifier of the primary investigator serving as the contact point for the related analysis record.
Expected Value: Text: ORCID Identifier
Controlled Terms: None
Deonticity: Recommended
Conditional Use: None
Number of Values Allowed: 0 – 1

Related Publication

Related Publication Citation

CSV Template Column Name: related_publication_citation
Description: The full bibliographic citation(s) for related publication(s) that use the data from this analysis.
Expected Value: Text
Controlled Terms: None
Deonticity: Optional
Conditional Use: None
Number of Values Allowed: 0+

Related Publication Identifier

CSV Template Column Name: related_publication_id
Description: The identifier for the selected ID type.
Expected Value: Text
Controlled Terms: None
Deonticity: Optional
Conditional Use: None
Number of Values Allowed: 0+

Related Publication Identifier Type

CSV Template Column Name: related_publication_id_type
Description: The type of digital identifier used for this publication (e.g., Digital Object Identifier (DOI)).
Expected Value: Text: Controlled Term

Controlled Terms: Required
Deonticity: Optional
Conditional Use: None
Number of Values Allowed: 0+
