# Supplementary material for: Design, development, and implementation of IsoBank: A centralized repository for isotopic data
Source: PLoS One. 2024 Sep 6;19(9):e0295662. doi: 10.1371/journal.pone.0295662 (PMC11379280; doi:10.1371/journal.pone.0295662)
Supplement: Peer review history

# Peer review: Decision Letter 0

**DOI:** 10.1371/journal.pone.0295662.r001

**Authors:** Daniel Becker

1 Aug 2023

PONE-D-23-13414Design, development, and implementation of IsoBank: a centralized repository for isotopic dataPLOS ONE

Dear Dr. Shipley,

Thank you for submitting your manuscript to PLOS ONE. After careful consideration, we feel that it has merit but does not fully meet PLOS ONE’s publication criteria as it currently stands. Therefore, we invite you to submit a revised version of the manuscript that addresses the points raised during the review process.

Please submit your revised manuscript by Sep 15 2023 11:59PM. If you will need more time than this to complete your revisions, please reply to this message or contact the journal office at plosone@plos.org. When you're ready to submit your revision, log on to https://www.editorialmanager.com/pone/ and select the 'Submissions Needing Revision' folder to locate your manuscript file.

Please include the following items when submitting your revised manuscript:

- A rebuttal letter that responds to each point raised by the academic editor and reviewer(s). You should upload this letter as a separate file labeled 'Response to Reviewers'.
- A marked-up copy of your manuscript that highlights changes made to the original version. You should upload this as a separate file labeled 'Revised Manuscript with Track Changes'.
- An unmarked version of your revised paper without tracked changes. You should upload this as a separate file labeled 'Manuscript'.

If you would like to make changes to your financial disclosure, please include your updated statement in your cover letter. Guidelines for resubmitting your figure files are available below the reviewer comments at the end of this letter.

If applicable, we recommend that you deposit your laboratory protocols in protocols.io to enhance the reproducibility of your results. Protocols.io assigns your protocol its own identifier (DOI) so that it can be cited independently in the future. For instructions see: https://journals.plos.org/plosone/s/submission-guidelines#loc-laboratory-protocols. Additionally, PLOS ONE offers an option for publishing peer-reviewed Lab Protocol articles, which describe protocols hosted on protocols.io. Read more information on sharing protocols at https://plos.org/protocols?utm_medium=editorial-email&utm_source=authorletters&utm_campaign=protocols.

We look forward to receiving your revised manuscript.

Kind regards,

Daniel Becker

Academic Editor

PLOS ONE

Journal Requirements:

When submitting your revision, we need you to address these additional requirements.

1. Please ensure that your manuscript meets PLOS ONE's style requirements, including those for file naming. The PLOS ONE style templates can be found at 

https://journals.plos.org/plosone/s/file?id=wjVg/PLOSOne_formatting_sample_main_body.pdf and 

https://journals.plos.org/plosone/s/file?id=ba62/PLOSOne_formatting_sample_title_authors_affiliations.pdf

2. Thank you for stating the following financial disclosure: 

"IsoBank is supported by National Science Foundation Grant #1759937."

Please state what role the funders took in the study.  If the funders had no role, please state: "The funders had no role in study design, data collection and analysis, decision to publish, or preparation of the manuscript." 

If this statement is not correct you must amend it as needed. 

Please include this amended Role of Funder statement in your cover letter; we will change the online submission form on your behalf.

3. Thank you for stating the following in the Acknowledgments Section of your manuscript: 

"We thank all community members that assisted with the design, implementation, and testing of

the IsoBank repository. IsoBank is supported by National Science Foundation Grant #1759937."

We note that you have provided funding information that is not currently declared in your Funding Statement. However, funding information should not appear in the Acknowledgments section or other areas of your manuscript. We will only publish funding information present in the Funding Statement section of the online submission form. 

Please remove any funding-related text from the manuscript and let us know how you would like to update your Funding Statement. Currently, your Funding Statement reads as follows: 

"IsoBank is supported by National Science Foundation Grant #1759937."

Please include your amended statements within your cover letter; we will change the online submission form on your behalf.

4. In your Data Availability statement, you have not specified where the minimal data set underlying the results described in your manuscript can be found. PLOS defines a study's minimal data set as the underlying data used to reach the conclusions drawn in the manuscript and any additional data required to replicate the reported study findings in their entirety. All PLOS journals require that the minimal data set be made fully available. For more information about our data policy, please see http://journals.plos.org/plosone/s/data-availability.

Upon re-submitting your revised manuscript, please upload your study’s minimal underlying data set as either Supporting Information files or to a stable, public repository and include the relevant URLs, DOIs, or accession numbers within your revised cover letter. For a list of acceptable repositories, please see http://journals.plos.org/plosone/s/data-availability#loc-recommended-repositories. Any potentially identifying patient information must be fully anonymized.

Important: If there are ethical or legal restrictions to sharing your data publicly, please explain these restrictions in detail. Please see our guidelines for more information on what we consider unacceptable restrictions to publicly sharing data: http://journals.plos.org/plosone/s/data-availability#loc-unacceptable-data-access-restrictions. Note that it is not acceptable for the authors to be the sole named individuals responsible for ensuring data access.

We will update your Data Availability statement to reflect the information you provide in your cover letter.

5. We note that Figure 3 in your submission contain map images which may be copyrighted. All PLOS content is published under the Creative Commons Attribution License (CC BY 4.0), which means that the manuscript, images, and Supporting Information files will be freely available online, and any third party is permitted to access, download, copy, distribute, and use these materials in any way, even commercially, with proper attribution. For these reasons, we cannot publish previously copyrighted maps or satellite images created using proprietary data, such as Google software (Google Maps, Street View, and Earth). For more information, see our copyright guidelines: http://journals.plos.org/plosone/s/licenses-and-copyright.

We require you to either (1) present written permission from the copyright holder to publish these figures specifically under the CC BY 4.0 license, or (2) remove the figures from your submission:

(1) You may seek permission from the original copyright holder of Figure 3 to publish the content specifically under the CC BY 4.0 license.  

We recommend that you contact the original copyright holder with the Content Permission Form (http://journals.plos.org/plosone/s/file?id=7c09/content-permission-form.pdf) and the following text:

“I request permission for the open-access journal PLOS ONE to publish XXX under the Creative Commons Attribution License (CCAL) CC BY 4.0 (http://creativecommons.org/licenses/by/4.0/). Please be aware that this license allows unrestricted use and distribution, even commercially, by third parties. Please reply and provide explicit written permission to publish XXX under a CC BY license and complete the attached form.”

Please upload the completed Content Permission Form or other proof of granted permissions as an ""Other"" file with your submission.

In the figure caption of the copyrighted figure, please include the following text: “Reprinted from [ref] under a CC BY license, with permission from [name of publisher], original copyright [original copyright year].”

(2) If you are unable to obtain permission from the original copyright holder to publish these figures under the CC BY 4.0 license or if the copyright holder’s requirements are incompatible with the CC BY 4.0 license, please either i) remove the figure or ii) supply a replacement figure that complies with the CC BY 4.0 license. Please check copyright information on all replacement figures and update the figure caption with source information. If applicable, please specify in the figure caption text when a figure is similar but not identical to the original image and is therefore for illustrative purposes only.

The following resources for replacing copyrighted map figures may be helpful:

USGS National Map Viewer (public domain): http://viewer.nationalmap.gov/viewer/

The Gateway to Astronaut Photography of Earth (public domain): http://eol.jsc.nasa.gov/sseop/clickmap/

Maps at the CIA (public domain): https://www.cia.gov/library/publications/the-world-factbook/index.html and https://www.cia.gov/library/publications/cia-maps-publications/index.html

NASA Earth Observatory (public domain): http://earthobservatory.nasa.gov/

Landsat: http://landsat.visibleearth.nasa.gov/

USGS EROS (Earth Resources Observatory and Science (EROS) Center) (public domain): http://eros.usgs.gov/#

Natural Earth (public domain): http://www.naturalearthdata.com/

6. Please review your reference list to ensure that it is complete and correct. If you have cited papers that have been retracted, please include the rationale for doing so in the manuscript text, or remove these references and replace them with relevant current references. Any changes to the reference list should be mentioned in the rebuttal letter that accompanies your revised manuscript. If you need to cite a retracted article, indicate the article’s retracted status in the References list and also include a citation and full reference for the retraction notice.

[Note: HTML markup is below. Please do not edit.]

Reviewers' comments:

Reviewer's Responses to Questions

**Comments to the Author**

1. Is the manuscript technically sound, and do the data support the conclusions?

The manuscript must describe a technically sound piece of scientific research with data that supports the conclusions. Experiments must have been conducted rigorously, with appropriate controls, replication, and sample sizes. The conclusions must be drawn appropriately based on the data presented. 

Reviewer #1: Yes

**********

2. Has the statistical analysis been performed appropriately and rigorously? 

Reviewer #1: N/A

**********

3. Have the authors made all data underlying the findings in their manuscript fully available?

The PLOS Data policy requires authors to make all data underlying the findings described in their manuscript fully available without restriction, with rare exception (please refer to the Data Availability Statement in the manuscript PDF file). The data should be provided as part of the manuscript or its supporting information, or deposited to a public repository. For example, in addition to summary statistics, the data points behind means, medians and variance measures should be available. If there are restrictions on publicly sharing data—e.g. participant privacy or use of data from a third party—those must be specified.

Reviewer #1: Yes

**********

4. Is the manuscript presented in an intelligible fashion and written in standard English?

PLOS ONE does not copyedit accepted manuscripts, so the language in submitted articles must be clear, correct, and unambiguous. Any typographical or grammatical errors should be corrected at revision, so please note any specific errors here.

Reviewer #1: Yes

**********

5. Review Comments to the Author

Please use the space provided to explain your answers to the questions above. You may also include additional comments for the author, including concerns about dual publication, research ethics, or publication ethics. (Please upload your review as an attachment if it exceeds 20,000 characters)

Reviewer #1: Shipley et al. provide an overview of the IsoBank repository for stable isotope data. As someone who generates and uses stable isotope data, I was aware of the IsoBank effort but have not engaged with it to date. Therefore, I appreciated this article. In terms of overall comments:

1. I would have liked to have read more in the article about how IsoBank plans to make itself sustainable to allow some of the future engagement/growth/development of the repository that is outlined in section 5. Please consider adding some text on this.

2. The IsoBank website seems out of date. The last update to the website was nearly 2 years ago (July 2021), there were only 6 datasets uploaded in 2022, and there is at least one inconsistency between this manuscript and the website (see below). These issues make me question how actively IsoBank is being developed, expanded, used, etc. It seems like the authors would want the website to be in tip top shape prior to the publication of this manuscript, so consider doing so. In that regard, it might be helpful to create videos for the website that provide an overview of IsoBank for potential users, as well as a deeper dive into IsoBank’s functionality.

3. Finally, the manuscript mentions legacy data. Such date are critical to archive before investigators retire, files are lost, etc. Is there going to be a push to get historical stable isotope data uploaded to IsoBank?

Specific comments

Abstract, 2nd sentence: Consider not using “grow” twice.

Abstract, 2nd sentence: I believe “continues” should be “continue”.

Abstract: “diverse users of isotopic data” is used twice. I’m not sure what is meant by “diverse” in this context and I’m not sure it is even necessary.

Intro, first sentence: Pauli et al. 2015a doesn’t seem like the most appropriate citation for this sentence.

Introduction, second paragraph, 3rd sentence: I believe “has” should be “have”.

Introduction, second paragraph, 4th sentence: I don’t believe all of these repositories are discipline specific. For example, isomemo.com says that it is “a Big Data initiative bringing together isotopic data from archaeology, ecology, and environmental & life sciences”, which clearly isn’t discipline specific. I would appreciate if the authors could clarify the gap that IsoBank aims to fill because at face value it seems like there a several options for archival of stable isotope data and it isn’t clear to me if/how they may be getting redundant with each other. It might also be helpful to provide guidance to potential submitters of data. For example, if I have a water stable isotope dataset should I upload it to IsoBank, waterisotopesdb.org, and/or Dryad? Or if I have a bone stable isotope dataset, should I upload it to IsoBank, Neotoma, or Dryad?

Page 5, last full sentence: As the authors know, there are a variety of unique challenges associated with QAQC of stable isotope data. They could briefly mention those here or simply provide a citation or two to relevant publications.

Page 6, first sentence of first full paragraph: What “information”?

Page 6, 3rd sentence of first full paragraph: What is meant by “walkthroughs” and “exercises”?

Page 6, 4th sentence of first full paragraph: “information” about what?

Page 6, third line from bottom: Please provide a citation for the “Darwin Core”.

Page 7: first two full sentences: Were these efforts at engagement successful?

Page 7: The text in the “Core features” section says that data can be either private or public. However, the Isobank website (https://isobank.tacc.utexas.edu/isobank-guide/) says “In future we will also allow you to limit access to these datasets to selected researchers, but for the time being all data uploaded to IsoBank is open to all.” Therefore, the website sounds like all data are made public. Which is correct? Also, I would start a new sentence after “…public datasets.”

Page 8, “Dataset Statistics”: It isn’t clear what “submitted datasets, analysis records, analysis metadata records, measurement records, and measurement metadata records” are. Please clarify.

Figure 5 legend: Are the colors, shapes, and direction of arrows meant to indicate anything particular?

Page 21: The “Of particular….” sentence could be moved to after the first sentence of this paragraph, because the “IsoBank wil be….” is an example of the “Of particular…” sentence.

**********

6. PLOS authors have the option to publish the peer review history of their article (what does this mean?). If published, this will include your full peer review and any attached files.

If you choose “no”, your identity will remain anonymous but your review may still be made public.

**Do you want your identity to be public for this peer review?** For information about this choice, including consent withdrawal, please see our Privacy Policy.

Reviewer #1: No

**********

[NOTE: If reviewer comments were submitted as an attachment file, they will be attached to this email and accessible via the submission site. Please log into your account, locate the manuscript record, and check for the action link "View Attachments". If this link does not appear, there are no attachment files.]

While revising your submission, please upload your figure files to the Preflight Analysis and Conversion Engine (PACE) digital diagnostic tool, https://pacev2.apexcovantage.com/. PACE helps ensure that figures meet PLOS requirements. To use PACE, you must first register as a user. Registration is free. Then, login and navigate to the UPLOAD tab, where you will find detailed instructions on how to use the tool. If you encounter any issues or have any questions when using PACE, please email PLOS at figures@plos.org. Please note that Supporting Information files do not need this step.

# Author comment: Author response to Decision Letter 0

**DOI:** 10.1371/journal.pone.0295662.r002

17 Nov 2023

Comment: Reviewer #1: Shipley et al. provide an overview of the IsoBank repository for stable isotope data. As someone who generates and uses stable isotope data, I was aware of the IsoBank effort but have not engaged with it to date. Therefore, I appreciated this article. 

Response: We are excited that the reviewer appreciated our manuscript, we have integrated all suggestions accordingly, which have greatly strengthened the paper. 

Comment: 1. I would have liked to have read more in the article about how IsoBank plans to make itself sustainable to allow some of the future engagement/growth/development of the repository that is outlined in section 5. Please consider adding some text on this.

Response: We have added new text at the beginning of section 5 that discusses potential mechanisms to achieve long-term financial sustainability. This was inspired through a recent report on database sustainability hosted by The Ecological Society of America (chrome-extension://efaidnbmnnnibpcajpcglclefindmkaj/https://esa.org/sbi/wp-content/uploads/2019/09/Workshop-Report_Sustaining-Data-Repositories_FINAL.pdf). 

This new section now reads (Lines 418 - 428) “These developments will largely focus on improvements to usability, which can be achieved through increased engagement with the scientific community and achieving a resilient financial model to support long-term sustainability. While submission of competitive grant proposals to funding bodies including, may provide short-term sustainability, more diverse and innovative models are required to ensure sustainability and resilience to financial instability over the long term. Strategies may include 1) subscription-based pricing, where users or institutions pay fees associated with the hosting of data, 2) integration of value-added services, which may include paying for advanced access to data tools or data tracking (i.e., statistics on how may users are utilizing uploaded datasets), 3) formal assignment of an institutional host that can provide reliable funding in return for credibility, and 4) curated marketing opportunities that require a premium.”

Comment: 2. The IsoBank website seems out of date. The last update to the website was nearly 2 years ago (July 2021), there were only 6 datasets uploaded in 2022, and there is at least one inconsistency between this manuscript and the website (see below). These issues make me question how actively IsoBank is being developed, expanded, used, etc. It seems like the authors would want the website to be in tip top shape prior to the publication of this manuscript, so consider doing so. In that regard, it might be helpful to create videos for the website that provide an overview of IsoBank for potential users, as well as a deeper dive into IsoBank’s functionality.

Response: During the revision process, the TACC team have undertaken a substantial rebuild of www.isobank.org. We have therefore updated all statistics, which are reflected in the manuscript. Regarding videos, we direct users to several online workshops that detail IsoBank’s history and provide guidance on the upload of data and querying the database. In the final section, we also provide proposed plans to increase the volume of online material that can assist with navigating IsoBank’s primary features (Lines 483 - 491). 

Comment: 3. Finally, the manuscript mentions legacy data. Such date are critical to archive before investigators retire, files are lost, etc. Is there going to be a push to get historical stable isotope data uploaded to IsoBank?

Response: We have included several new sentences in section 5, that underscores the importance of ingesting legacy datasets, to support big data compilations. Our new text now reads (Lines 517 - 525) “As IsoBank grows in terms of the type and number of datasets, so will the capacity for cross-disciplinary big data initiatives and integration, which are critical for supporting highly impactful scientific studies of global relevance [36, 37] and development of novel research proposals. Given the high volume of published studies reporting isotope measurements, integration of historical legacy data must be a core focus of IsoBank’s future development. We anticipate that efforts to compile and upload legacy data could be proposed and implemented as funded projects by disciplinary investigators. This would support impactful macroscale research, such as development of novel isoscapes and refinement of global biogeochemical models (e.g., hydrology, carbon cycling, nitrogen cycling etc).”

Specific Comments

Comment: Abstract, 2nd sentence: Consider not using “grow” twice.

Response: We have implemented this suggestion. 

Comment: Abstract, 2nd sentence: I believe “continues” should be “continue”.

Response: We have implemented this suggestion. 

Comment: Abstract: “diverse users of isotopic data” is used twice. I’m not sure what is meant by “diverse” in this context and I’m not sure it is even necessary.

Response: We have removed both mentions of the term ‘diverse users’. We agree that the term is too vague. 

Comment: Intro, first sentence: Pauli et al. 2015a doesn’t seem like the most appropriate citation for this sentence.

Response: We agree that the Pauli reference is not the most appropriate. We have removed this citation from the sentence. 

Comment: Introduction, second paragraph, 3rd sentence: I believe “has” should be “have”.

Response: We have implemented this suggestion. 

Comment: Introduction, second paragraph, 4th sentence: I don’t believe all of these repositories are discipline specific. For example, isomemo.com says that it is “a Big Data initiative bringing together isotopic data from archaeology, ecology, and environmental & life sciences”, which clearly isn’t discipline specific. I would appreciate if the authors could clarify the gap that IsoBank aims to fill because at face value it seems like there a several options for archival of stable isotope data and it isn’t clear to me if/how they may be getting redundant with each other. 

Response: We agree with the reviewer that the initial submission did not provide a clear rationale for the gap that IsoBank intends to fill. As such, we have rewritten this section as follows (lines 159-167) “However, the stable isotope research community has lacked a globally available, cross-disciplinary data repository with unifying metadata fields. This has limited development of big data isotope-based research and restricted the potential of isotopic information to facilitate systems-based thinking. For example, ecological studies have shown that combining isotopic information from avian feathers and freshwater bodies can permit the tracking of bird migration patterns [18]. But isotopic records from animals and from the hydrosphere are typically preserved in distinct archives whose metadata customs differ substantially. By creating a repository in which isotopic information from diverse disciplines is normalized through a unifying data and metadata template, IsoBank facilitates vast opportunities for novel scientific discovery.”

Comment: It might also be helpful to provide guidance to potential submitters of data. For example, if I have a water stable isotope dataset should I upload it to IsoBank, waterisotopesdb.org, and/or Dryad? Or if I have a bone stable isotope dataset, should I upload it to IsoBank, Neotoma, or Dryad?

Response: While it is our intention to promote the use of IsoBank, the decision on which repository to use ultimately falls with the user. Our manuscript intends to provide rationale as to why one may consider submitting data to IsoBank, but we do not wish to discount the utility of other discipline-specific databases. In the final section of our manuscript, we describe goals for expanding IsoBank beyond a data repository, exclusively, that details a plan to establish pipelines between other data repositories. Thus, in the future we envisage that data submitted to one repository may be shared and available through all, with IsoBank as the catalyst and/or exchange platform. 

Comment: Page 5, last full sentence: As the authors know, there are a variety of unique challenges associated with QAQC of stable isotope data. They could briefly mention those here or simply provide a citation or two to relevant publications.

Response: We have now provided two additional citations (Jardine and Cunjak 2005; Paul et al. 2007) to support the statement relating to QAQC. 

Jardine, T. D., & Cunjak, R. A. (2005). Analytical error in stable isotope ecology. Oecologia, 144, 528-533.

Paul, D., Skrzypek, G., & Fórizs, I. (2007). Normalization of measured stable isotopic compositions to isotope reference scales–a review. Rapid Communications in Mass Spectrometry: An International Journal Devoted to the Rapid Dissemination of Up‐to‐the‐Minute Research in Mass Spectrometry, 21(18), 3006-3014.

Comment: Page 6, first sentence of first full paragraph: What “information”?

Comment: Page 6, 3rd sentence of first full paragraph: What is meant by “walkthroughs” and “exercises”?

Comment: Page 6, 4th sentence of first full paragraph: “information” about what?

Response: We agree that this section was unclear and have integrated the above three comments. We have provided additional clarity in this text, which now reads (lines 211 - 218) “These conversations were guided by interactive walkthroughs of the working metadata schema led by the executive committee (i.e., DSSC leads). Committee-specific online workspaces were created in google drive that contained editable, shared documents for DSSC members to express needs, write notes, and capture comments. Following breakout sessions, DSSCs rejoined a group session and reported on their discussions and decisions. In this setting, all members had the opportunity to determine where needs overlapped and diverged, and then to reach a consensus in a group conversation.”

Comment: Page 6, third line from bottom: Please provide a citation for the “Darwin Core”.

Response: We have now provided hyperlinks and citations associated with Darwin Core (https://dwc.tdwg.org, Wieczorek et al. 2012). 

Wieczorek, J., Bloom, D., Guralnick, R., Blum, S., Döring, M., Giovanni, R., ... & Vieglais, D. (2012). Darwin Core: an evolving community-developed biodiversity data standard. PloS one, 7(1), e29715

Comment: Page 7: first two full sentences: Were these efforts at engagement successful?

Response: We note that the first engagement efforts were highly successful, with a 40-person online workshop run through IsoEcol in Spring of 2021. 

Comment: Page 7: The text in the “Core features” section says that data can be either private or public. However, the Isobank website (https://isobank.tacc.utexas.edu/isobank-guide/) says “In future we will also allow you to limit access to these datasets to selected researchers, but for the time being all data uploaded to IsoBank is open to all.” Therefore, the website sounds like all data are made public. Which is correct? Also, I would start a new sentence after “…public datasets.”

Response: We agree that this section was unclear, and we have therefore provided additional clarity on what we consider to be public versus private, with regards to data access – this information is summarized in a new Table (Table 1). This new section now reads (lines 238 – 249) “The IsoBank system is built on a PostgreSQL database with a web interface developed using the Django framework. Many IsoBank features, such as accessing metadata templates, are publicly accessible and do not require a user account. To be sensitive to researcher challenges with data sharing and open access policies, registered users can choose to upload datasets that are either private (i.e., can only be viewed and queried by the uploader with no embargo period) or can be accessed by any registered user. Storing private data on IsoBank aids users in 1) general data management and/or fulfilling funder requirements for data management and 2) ease of publication and/or making data publicly available at the appropriate time. User registration was initially facilitated by providing an email address and password; however, the system now requires manual approval of new users by administrators to filter non-authentic users (e.g., bots). Below we provide further information on the features that can be accessed at public, registered user, and administrator levels (Table 1).”

Comment: Page 8, “Dataset Statistics”: It isn’t clear what “submitted datasets, analysis records, analysis metadata records, measurement records, and measurement metadata records” are. Please clarify.

Response: We have now provided a new table (Table 2) that defines each of the presented statistics. 

Comment: Figure 5 legend: Are the colors, shapes, and direction of arrows meant to indicate anything particular?

Response: We have improved clarity within the revised figure caption, which now reads “Figure 5. Proposed vision for continued development of community engagement initiatives for IsoBank (maroon boxes), including outreach, journal/publisher partnerships, technological additionality, and facilitation of big data compilations. Gray boxes represent specific activities that improve each engagement initiative.”

Comment: Page 21: The “Of particular….” sentence could be moved to after the first sentence of this paragraph, because the “IsoBank wil be….” is an example of the “Of particular…” sentence.

Response: We have implemented this change as suggested.

# Peer review: Decision Letter 1

**DOI:** 10.1371/journal.pone.0295662.r003

**Authors:** Daniel Becker

28 Nov 2023

Design, development, and implementation of IsoBank: a centralized repository for isotopic data

PONE-D-23-13414R1

Dear Dr. Shipley,

We’re pleased to inform you that your manuscript has been judged scientifically suitable for publication and will be formally accepted for publication once it meets all outstanding technical requirements.

Within one week, you’ll receive an e-mail detailing the required amendments. When these have been addressed, you’ll receive a formal acceptance letter and your manuscript will be scheduled for publication.

An invoice for payment will follow shortly after the formal acceptance. To ensure an efficient process, please log into Editorial Manager at http://www.editorialmanager.com/pone/, click the 'Update My Information' link at the top of the page, and double check that your user information is up-to-date. If you have any billing related questions, please contact our Author Billing department directly at authorbilling@plos.org.

If your institution or institutions have a press office, please notify them about your upcoming paper to help maximize its impact. If they’ll be preparing press materials, please inform our press team as soon as possible -- no later than 48 hours after receiving the formal acceptance. Your manuscript will remain under strict press embargo until 2 pm Eastern Time on the date of publication. For more information, please contact onepress@plos.org.

Kind regards,

Daniel Becker

Academic Editor

PLOS ONE

Additional Editor Comments (optional):

Reviewers' comments:

Reviewer's Responses to Questions

**Comments to the Author**

1. If the authors have adequately addressed your comments raised in a previous round of review and you feel that this manuscript is now acceptable for publication, you may indicate that here to bypass the “Comments to the Author” section, enter your conflict of interest statement in the “Confidential to Editor” section, and submit your "Accept" recommendation.

Reviewer #1: All comments have been addressed

**********

2. Is the manuscript technically sound, and do the data support the conclusions?

The manuscript must describe a technically sound piece of scientific research with data that supports the conclusions. Experiments must have been conducted rigorously, with appropriate controls, replication, and sample sizes. The conclusions must be drawn appropriately based on the data presented. 

Reviewer #1: Yes

**********

3. Has the statistical analysis been performed appropriately and rigorously? 

Reviewer #1: Yes

**********

4. Have the authors made all data underlying the findings in their manuscript fully available?

The PLOS Data policy requires authors to make all data underlying the findings described in their manuscript fully available without restriction, with rare exception (please refer to the Data Availability Statement in the manuscript PDF file). The data should be provided as part of the manuscript or its supporting information, or deposited to a public repository. For example, in addition to summary statistics, the data points behind means, medians and variance measures should be available. If there are restrictions on publicly sharing data—e.g. participant privacy or use of data from a third party—those must be specified.

Reviewer #1: Yes

**********

5. Is the manuscript presented in an intelligible fashion and written in standard English?

PLOS ONE does not copyedit accepted manuscripts, so the language in submitted articles must be clear, correct, and unambiguous. Any typographical or grammatical errors should be corrected at revision, so please note any specific errors here.

Reviewer #1: Yes

**********

6. Review Comments to the Author

Please use the space provided to explain your answers to the questions above. You may also include additional comments for the author, including concerns about dual publication, research ethics, or publication ethics. (Please upload your review as an attachment if it exceeds 20,000 characters)

Reviewer #1: Thanks for your thorough responses. Just a few very minor editorial revisions:

Line 120: there is a period missing after “2021”.

Line 336: please delete the extra comma.

Line 397: please change “has” to “have”

Line 495: I believe the second “and” in this sentence should be deleted

**********

7. PLOS authors have the option to publish the peer review history of their article (what does this mean?). If published, this will include your full peer review and any attached files.

If you choose “no”, your identity will remain anonymous but your review may still be made public.

**Do you want your identity to be public for this peer review?** For information about this choice, including consent withdrawal, please see our Privacy Policy.

Reviewer #1: No

**********

# Editor report: Acceptance letter

**DOI:** 10.1371/journal.pone.0295662.r004

**Authors:** Daniel Becker

12 Jun 2024

PONE-D-23-13414R1 

PLOS ONE

Dear Dr. Shipley, 

I'm pleased to inform you that your manuscript has been deemed suitable for publication in PLOS ONE. Congratulations! Your manuscript is now being handed over to our production team.

At this stage, our production department will prepare your paper for publication. This includes ensuring the following:

* All references, tables, and figures are properly cited

* All relevant supporting information is included in the manuscript submission,

* There are no issues that prevent the paper from being properly typeset

If revisions are needed, the production department will contact you directly to resolve them. If no revisions are needed, you will receive an email when the publication date has been set. At this time, we do not offer pre-publication proofs to authors during production of the accepted work. Please keep in mind that we are working through a large volume of accepted articles, so please give us a few weeks to review your paper and let you know the next and final steps. 

Lastly, if your institution or institutions have a press office, please let them know about your upcoming paper now to help maximize its impact. If they'll be preparing press materials, please inform our press team within the next 48 hours. Your manuscript will remain under strict press embargo until 2 pm Eastern Time on the date of publication. For more information, please contact onepress@plos.org.

If we can help with anything else, please email us at customercare@plos.org.

Thank you for submitting your work to PLOS ONE and supporting open access. 

Kind regards, 

PLOS ONE Editorial Office Staff

on behalf of

Daniel Becker 

Academic Editor

PLOS ONE
